# *Drosophila* storage proteins promote both the rate and the duration of tumor growth

**DOI:** 10.1126/sciadv.aeb2956

**Published:** 2026-07-03

**Authors:** Luca Valzania, Dalmiro Blanco-Obregon, Aya Alami, Pierre Léopold

**Affiliations:** CNRS UMR3215, INSERM U934, Institut Curie, PSL Research University, UPMC Paris-Sorbonne, 26 rue d’Ulm, 75005 Paris, France.

## Abstract

Amino acid storage proteins, such as serum albumin in mammals, are known to accumulate within tumors, but their precise contribution to tumor biology remains unclear. Using both cachectic and noncachectic tumor models in *Drosophila*, we observed that fat body–derived hexamerins accumulate in the tumors through a selective uptake process. Disabling this uptake led to a marked reduction in tumor growth, demonstrating that hexamerins are used as a nutrient source to support cancer progression. Hexamerin uptake also supports expression of the relaxin-like *Drosophila* insulin-like protein 8 (Dilp8) by the tumor, inhibiting ecdysone production and extending the growth period. This coupling between nutrient uptake by the tumor and inhibition of the developmental progression exerts a full diversion of the host resources. Functional parallels with mammalian albumins suggest evolutionarily conserved mechanisms with potential implications for cancer biology.

## Teaser

*Drosophila* tumors hijack storage proteins to grow faster and extend their growth window by blocking host development.

## INTRODUCTION

A fundamental challenge faced by rapidly growing tumors is to secure sufficient nutrients to support their relentless anabolic demands. Since Warburg’s early recognition of altered carbohydrate metabolism in cancer cells (*1*), a wide range of low molecular weight nutrients, including fatty acids, ketone bodies, and amino acids, have been investigated as critical contributors to tumor growth and survival. However, despite decades of study, a comprehensive understanding of tumor nutrient metabolism and energy requirements remains elusive (*2*). As a result, identifying additional nutrient sources that support tumor proliferation continues to be a major focus in cancer research.

One historically underexplored nutrient source is plasma proteins. As early as 1948, Mider *et al.* (*3*) suggested that plasma proteins such as serum albumin might serve as nitrogen donors for tumors. Subsequent studies confirmed that albumin can accumulate in the tumor microenvironment and be taken up by cancer cells (*4*). Nevertheless, the concept of plasma protein catabolism as a direct amino acid supply for proliferating tumor cells has not been widely investigated. In parallel, the use of albumin as a drug carrier in cancer therapy has attracted increasing attention over the past two decades, highlighting its biological importance in tumors (*5*). Despite this interest, the molecular players mediating albumin uptake, such as specific receptors and binding proteins, remain poorly characterized (*6*).

In mammals, a major obstacle to investigating albumin’s role in tumor biology is its constitutive synthesis throughout life. By contrast, insects offer a tractable model to study storage proteins with precise temporal resolution. In these organisms, functional homologs of albumin are represented by hexamerins, which share multiple key features with mammalian serum albumin. They are the most abundant proteins in the circulatory system, they are primarily synthesized by an organ analogous to the liver, they act as dynamic reservoirs of amino acids, they support metabolic demands throughout the life cycle, they bind and transport small hydrophobic molecules and hormones, and their synthesis is tightly regulated by hormonal and nutritional cues (*7*). Because of this ensemble of functional similarities, hexamerins provide a unique opportunity to investigate how storage proteins contribute to tumor metabolism. In *Drosophila melanogaster*, there are two hexamerins [Larval Serum Proteins 1 and 2 (LSP1 and LSP2)] and two associated proteins [Fat Body Proteins 1 and 2 (FBP1 and FBP2)] (*7*). These storage proteins are synthesized by the fat body during a defined window of larval development and accumulate in the hemolymph. Subsequently, LSPs and FBP2 are reimported into the fat body via FBP1, where they serve as essential nutrient reserves for metamorphosis (*8*).

*Drosophila* offers powerful genetics to manipulate both tumor cells and host tissues, enabling a detailed, tissue-specific analysis of nutrient exchanges. While prior studies have revealed tumor-induced metabolic rewiring and systemic wasting reminiscent of cancer cachexia in flies (*9*), the role of macromolecular nutrients such as hexamerins has not been explored. In this study, we identify a mechanism by which epithelial tumors in *Drosophila* larvae hijack hexamerin resources to promote their own growth. We show that tumors actively internalize hexamerins through FBP1 and that loss of FBP1 impairs hexamerin uptake and limits tumor expansion. In addition to nutrient hijacking, tumors exert profound systemic effects on their hosts, including developmental delay, metabolic dysfunction, and cachexia-like wasting. In *Drosophila*, epithelial tumors can secrete the relaxin-like hormone *Drosophila* insulin-like protein 8 (Dilp8), which coordinates tissue growth and delays metamorphosis in response to local stress or growth perturbations (*10*–*14*). Here, we demonstrate that hexamerin uptake by tumors is required to induce *dilp8* expression and delay pupariation, thereby providing tumors with extended time for growth.

Using *Drosophila* to dissect nutrient fluxes between the host and the tumor, our work uncovers a tumor-driven strategy to best exploit host storage proteins and the developmental time window for growth.

## RESULTS

### Fat body–derived hexamerins accumulate in Yki^S168A^ tumors

To investigate the role of amino acid storage proteins in a tumor context, we used a *Drosophila* model of epithelial tumorigenesis based on the tissue-specific overexpression of a constitutively active form of Yorkie (*UAS-yki^S168A^-gfp*), the fly homolog of the mammalian oncogene Yes-associated protein (YAP) (*15*, *16*). Expression was driven in the wing imaginal disc using the *pdm2-Gal4* driver. This manipulation overrides Hippo pathway inhibition, resulting in uncontrolled tissue growth and a failure to initiate pupariation (fig. S1A). As a consequence, tumor-bearing larvae enter an extended larval stage lasting ~8 days and ultimately die without forming pupae (fig. S1A). These tumors, which initiate during the third larval instar, expand significantly and reach volumes up to 15 times larger than those of *wild-type* wing discs (*17*). Notably, this developmental window coincides with the peak of hexamerin production (*8*), making this model well suited for studying tumor/albumin-like protein interactions.

**Fig. 1.**
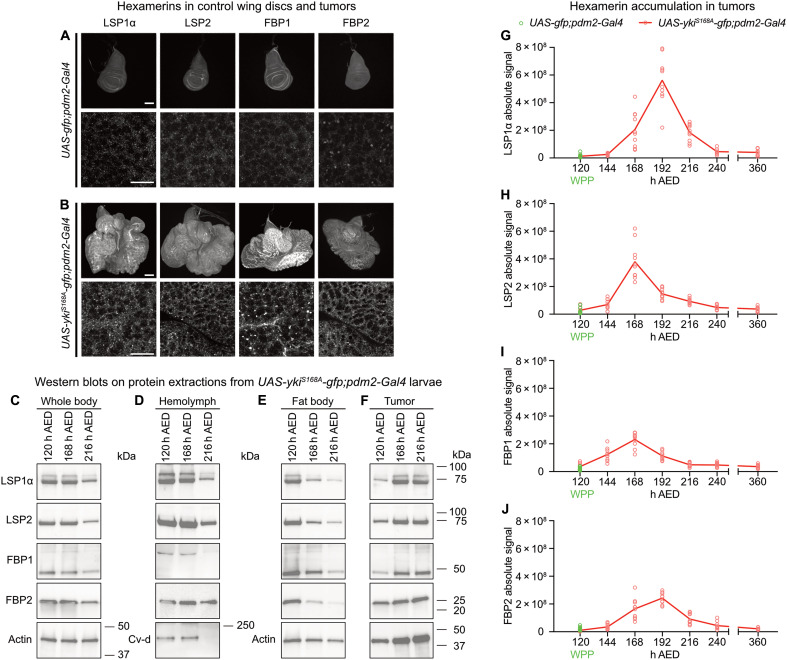
Yki^S168A^ tumors sequester circulating hexamerins, diverting them from normal reuptake by the fat body. (**A**) Representative wing imaginal discs dissected at 120 hours after egg deposition (AED) from *UAS-gfp;pdm2-Gal4* white prepupae (WPP), stained with antibodies against LSP1α, LSP2, FBP1, and FBP2. Top row: Entire discs shown; scale bar, 100 μm. Bottom row: High-magnification views of the wing pouch show hexamerins localized inside disc epithelial cells; scale bar, 10 μm. (**B**) Representative tumors from wing discs dissected from *UAS-yki^S168A^-gfp;pdm2-Gal4* larvae stained with the same antibodies. Top row: Full tumor views; scale bar, 100 μm. Bottom row: High-magnification images show robust hexamerin accumulation within tumor cells; scale bar, 10 μm. (**C** to **F**) Immunoblots of protein extracts from whole body (C), hemolymph (D), fat body (E), and tumors (F) of *UAS-yki^S168A^-gfp;pdm2-Gal4* larvae collected at indicated time points (hours AED). Blots were probed with antibodies against LSP1α, LSP2, FBP1, FBP2, and loading controls Actin and Crossveinless-d (Cv-d). Molecular weight markers are indicated on the right. h, hours. (**G** to **J**) Quantification of LSP1α (G), LSP2 (H), FBP1 (I), and FBP2 (J) levels through immunostaining in tumors during the extended third instar stage of *UAS-yki^S168A^-gfp;pdm2-Gal4* larvae. Time points (in hours AED) are shown on the *x* axis. The 120 hours AED time point corresponds to the pupariation of *UAS-gfp;pdm2-Gal4* control larvae, from which hexamerin levels were quantified in wing disc pouch. For each time point, 10 individual discs or tumors were analyzed.

We started asking whether these tumors accumulate hexamerins, similar to the accumulation of albumin and other nutrient carriers observed in some mammalian cancers. To this end, we used custom-generated antibodies against LSPs and FBPs (*8*) to perform immunofluorescence staining on *UAS-yki^S168A^-gfp;pdm2-Gal4* tumor tissue. Although hexamerin presence in the wing imaginal discs lacking tumors was consistently very low and may reflect either minimal physiological levels or background antibody staining (Fig. 1A), we observed a notable accumulation of both LSPs and FBPs in tumor-bearing discs (Fig. 1B, top row). The signal was particularly intense at tissue folds, raising the possibility of nonspecific trapping of the antibodies. However, high-resolution confocal microscopy revealed that hexamerins were localized in vesicular structures within the cytoplasm of tumor cells (Fig. 1B, bottom row), commonly viewed as a sign of intracellular uptake.

To determine the origin of the hexamerins accumulating in tumors, we first compared the mRNA levels of all hexamerin-encoding genes in wing imaginal discs and fat body tissue. As expected, expression of these genes was high in the fat body and very low in *wild-type* wing discs (fig. S1B), consistent with the fat body being the primary site of hexamerin synthesis (*8*). In wing discs from tumor-bearing larvae, expression was even further reduced, ruling out local production by the tumor as the source of hexamerin accumulation (fig. S1C). We next analyzed hexamerin gene expression in fat body tissue from control (*UAS-gfp;pdm2-Gal4*) and tumor-bearing (*UAS-yki^S168A^-gfp;pdm2-Gal4*) animals to assess whether hexamerin production is modified by the presence of the tumors. We found no significant changes in expression of *Lsp1* (fig. S1, D and E), *Lsp2* (fig. S1, F and G), or *Fbp2* (fig. S1, J and K) between the two conditions. The only notable difference was a reduction in *Fbp1* expression in *UAS-yki^S168A^-gfp;pdm2-Gal4* larvae at 128 hours after egg deposition (AED) (fig. S1, H and I). This is consistent with a reduced production of ecdysone, the major steroid hormone regulating insect development, in these animals (*18*–*20*) and with the fact that *Fbp1* expression relies, in part, on ecdysone (*8*).

We next investigated how hexamerin distribution changes during tumor progression. To do this, we performed Western blot analyses on protein extracts from whole larvae, hemolymph, dissected fat body, and tumor tissue at multiple time points throughout the extended larval stage (for details, see Materials and Methods). In tumor-bearing animals, total hexamerin levels in whole-body extracts decreased progressively over time (Fig. 1C; see normalization to actin levels in fig. S1L). A similar decrease was observed in the hemolymph [Fig. 1D and fig. S1M; hemolymph levels are normalized to Cv-d (*21*); however, this lipoprotein is no longer detectable at 216 hours AED precluding normalization for this time point] and in the fat body (Fig. 1E and fig. S1N). By contrast, we observed a progressive increase of hexamerin signals in the tumor paralleled with an increase in actin levels, reflecting the presence of elevated hexamerin levels during the first phase of tumor growth (Fig. 1F and fig. S1O).

To further evaluate the dynamics of hexamerin levels in the tumor, we developed a custom Fiji macro (see Materials and Methods) to measure hexamerin immunofluorescence from full confocal Z-stacks. The integration of the fluorescent signal over entire tumors revealed that hexamerin levels increase during the first 2 to 3 days of the extended larval period and then start declining (Fig. 1, G to J). Collectively, these findings indicate that hexamerins are produced by the fat body, secreted into the hemolymph and subsequently taken up by tumor cells.

### FBP1-dependent uptake of hexamerins enables tumor growth

We next sought to determine the mechanism by which hexamerins are taken up by tumors. In the normal developmental context, hexamerins enter the fat body only when bound to a specific serum protein called FBP1, which is produced by fat cells at the onset of metamorphosis (*8*). This raised the question of whether FBP1 is also required for hexamerin uptake by tumors.

To address this, our initial strategy was to use two independent binary expression systems: the LexA/LexAop system to induce tumor formation in the wing disc and the Gal4/UAS system to down-regulate *Fbp1* expression in the fat body. For this, we used a *LexAop-Yki^3S/A^* strain previously reported to generate gut tumors in adult *Drosophila* (*22*). However, this strain did not induce detectable tumors when crossed with either *pdm2-LexA* or *rn-LexA*, both targeting the larval wing disc. Given this limitation, we adopted an alternative approach using two Gal4/UAS systems to simultaneously induce Yki^S168A^-driven tumor growth in the wing disc and silence *Fbp1* expression in the fat body. Because this approach results in ectopic Yki activation in the fat body and *Fbp1* knockdown in the wing disc, we performed a set of control experiments to rule out unintended developmental effects that could confound our interpretation. Specifically, we assessed larval feeding behavior during the third instar (fig. S2A), wing disc volume (fig. S2B), hexamerin expression at 120 hours AED (fig. S2, C to H), timing of pupariation (fig. S2I), eclosion (fig. S2J), and adult wing size (fig. S2K) across all relevant single- and double-driver combinations, as well as coexpression of the two UAS transgenes with each driver individually. These analyses did not reveal phenotypes caused by ectopic Yki activation in the fat body or *Fbp1* knockdown in the tumor, except for a slight delay at pupal eclosion and a mild reduction of adult wing size (fig. S2, J and K) as previously reported for *Fbp1* depletion in the fat body (*8*).

**Fig. 2.**
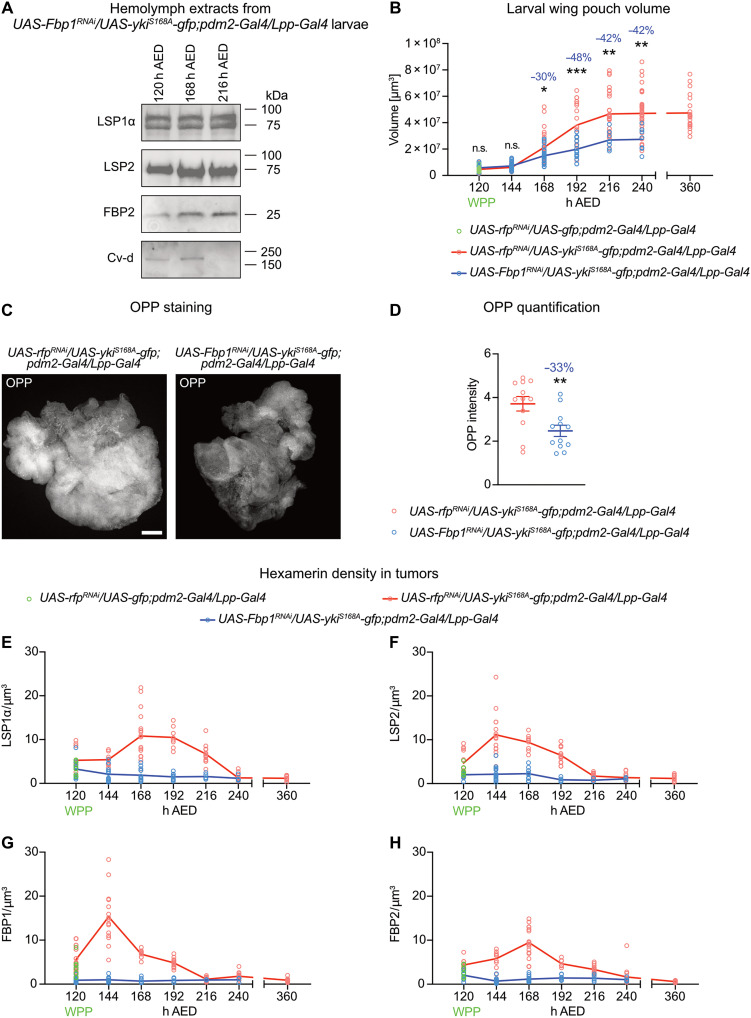
Hexamerins enter Yki^S168A^ tumors via an FBP1-dependent mechanism and support tumor growth. (**A**) Immunoblots of hemolymph extracts from *UAS-Fbp1^RNAi^/UAS-yki^S168A^-gfp;pdm2-Gal4/Lpp-Gal4* larvae at the indicated time points. Antibodies and molecular weight markers are shown to the left and to the right, respectively. (**B**) Quantification of wing pouch volume in tumor-bearing larvae, either in the presence or absence of FBP1. Volumes were measured at the indicated time points. Wing pouch volume of control larvae (*UAS-rfp^RNAi^/UAS-gfp;pdm2-Gal4/Lpp-Gal4*) was analyzed at pupariation (120 hours AED). A total of 7 to 38 wing pouches was analyzed per genotype at each time point. Statistical significance was assessed using unpaired *t* tests or one-way analysis of variance (ANOVA), as appropriate. n.s., not significant; *P ≤ 0.05; **P ≤ 0.01; ***P ≤ 0.001. The percentage reduction in wing pouch volume in the FBP1-depleted condition relative to *UAS-rfp^RNAi^/UAS-yki^S168A^-gfp;pdm2-Gal4/Lpp-Gal4* is indicated above the asterisks. No surviving *UAS-Fbp1^RNAi^/UAS-yki^S168A^-gfp;pdm2-Gal4/Lpp-Gal4* larvae were observed at 360 hours AED. (**C**) Confocal micrographs showing tumor wing discs from *UAS-rfp^RNAi^/UAS-yki^S168A^-gfp;pdm2-Gal4/Lpp-Gal4* and *UAS-Fbp1^RNAi^/UAS-yki^S168A^-gfp;pdm2-Gal4/Lpp-Gal4* larvae, visualizing the incorporation of OPP. Scale bar, 100 μm. (**D**) Quantitative analysis of OPP fluorescence in tumor wing discs from larvae expressing or lacking FBP1. Twelve discs from each genotype were examined. Statistical differences were assessed using an unpaired *t* test with Welch’s correction, with significance indicated as **P ≤ 0.01. The value above the asterisks denotes the percentage decrease in OPP incorporation caused by FBP1 depletion compared with control. (**E** to **H**) Quantification of intratumoral density of LSP1α (E), LSP2 (F), FBP1 (G), and FBP2 (H) during the prolonged third instar stage in larvae of the following genotypes: *UAS-rfp^RNAi^/UAS-yki^S168A^-gfp;pdm2-Gal4/Lpp-Gal4* and *UAS-Fbp1^RNAi^/UAS-yki^S168A^-gfp;pdm2-Gal4/Lpp-Gal4*. For the 120 hours AED reference time point, corresponding to pupariation in *UAS-rfp^RNAi^/UAS-gfp;pdm2-Gal4/Lpp-Gal4* WPP, hexamerin levels were measured in the wing disc pouch and normalized by pouch volume. A total of 3 to 24 individual discs or tumors was analyzed per condition and time point.

We then performed immunofluorescence quantification of hexamerins in tumors in the presence or absence of FBP1. Notably, *Fbp1* expression, already at very low levels in the wing disc, further declined over time in tumor-bearing discs (fig. S3A), suggesting that hexamerin uptake in the tumor is not driven by local *Fbp1* expression. While in control tumor-bearing larvae, hexamerins robustly accumulated in tumor tissues (fig. S3, B to E), they were barely detectable upon *Fbp1* knockdown (fig. S3, B to E). Western blot analysis on hemolymph samples from *UAS-Fbp1^RNAi^/UAS-yki^S168A^-gfp;pdm2-Gal4/Lpp-Gal4* larvae showed sustained storage protein levels (Fig. 2A, to compare with Fig. 1D), suggesting that FBP1 depletion, by preventing hexamerin uptake both in fat body and in tumor cells, induces their accumulation in the hemolymph.

To evaluate the functional importance of hexamerin uptake in supporting tumor growth, we performed volumetric reconstructions of tumors using confocal microscopy combined with three-dimensional (3D) image analysis. In control tumor-bearing larvae, tumor volume increases rapidly between 144 and 192 hours AED, then growth slows down, and tumors reach a plateau that persists until larval death (Fig. 2B). In contrast, tumors from animals lacking *Fbp1* expression were significantly smaller at all time points starting from 168 hours AED (Fig. 2B), suggesting that albumin-like protein uptake by the tumor is required for its expansion. Consistent with this interpretation, the tumor growth rate closely mirrored the temporal profile of hexamerin accumulation within the tumors (fig. S3F). To determine whether internalized hexamerins directly contribute to tumor growth, we quantified protein synthesis in tumors using O-propargyl-puromycin (OPP) incorporation assays, which serve as a proxy for global translational activity (Fig. 2C). Tumors deprived of hexamerins exhibited 33% reduction in protein synthesis relative to control tumors (Fig. 2D), indicating that hexamerin complexes are used as an anabolic resource to sustain tumor biosynthetic activity and growth. Although hexamerins present in the tumor constitute an autonomous amino acid resource to tumor cells, it is also possible that hexamerins taken up by fat body cells followed by their breakdown into amino acids also indirectly contribute to tumor growth.

We then used tumor volume data to estimate the relative density of storage proteins within the tumor over time. We observed that intratumor hexamerin concentrations increased during the first 24 to 48 hours of the extended larval stage, after which they dropped markedly (Fig. 2, E to H). This decline could reflect the combined effect of a progressive increase in tumor mass and the gradual depletion of circulating hexamerins, as the fat body stops producing them.

Together, these findings indicate that FBP1-mediated uptake of albumin-like proteins provides nutrient to the tumor and that this process plays a critical role in fueling tumor growth.

### Cachectic and noncachectic tumors reallocate hexamerins from the fat body to sustain their growth

Notably, the Yki^S168A^ tumor is known to induce a cachexia-like syndrome, characterized by systemic wasting of fat and muscle tissues (*9*, *23*). Cachexia has been proposed to support tumor development by providing nutrients derived from host tissue breakdown (*9*, *18*, *24*–*26*). This raised the possibility that, in the context of cachexia, hexamerins might be redirected to the tumor due to impaired uptake by the fat body.

**Fig. 3.**
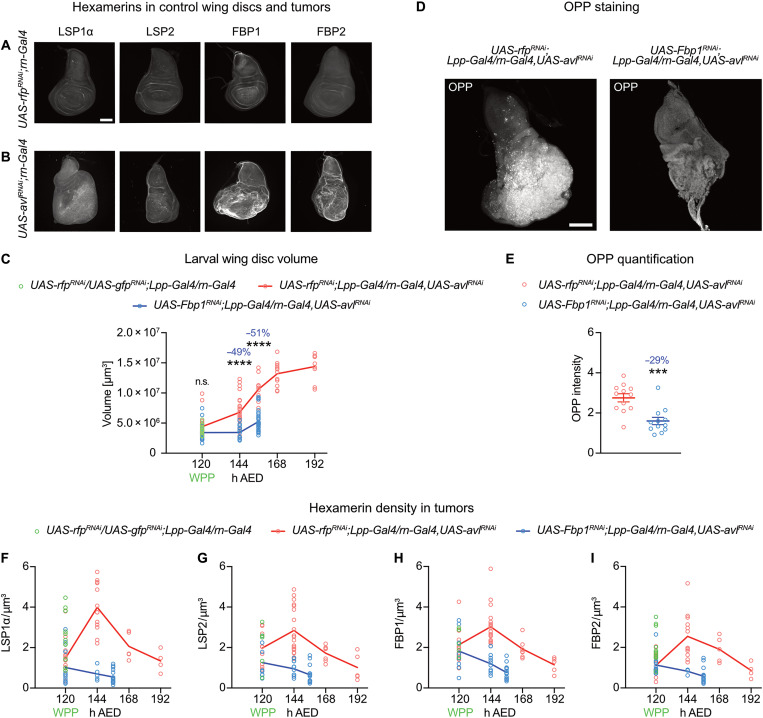
Hexamerins are taken up by *avl^RNAi^* tumors through FBP1 action and contribute to tumor growth. (**A** and **B**) Representative images of wing imaginal discs from control animals (A) and from *UAS-avl^RNAi^;rn-Gal4* larvae (B), stained with antibodies against LSP1α, LSP2, FBP1, and FBP2. Scale bar, 100 μm. (**C**) Quantification of wing disc volume in *avl^RNAi^* tumor-bearing larvae, either with or without FBP1. Disc volumes were measured at the indicated time points (in hours AED). As a reference, control discs from *UAS-rfp^RNAi^/UAS-gfp^RNAi^;Lpp-Gal4/rn-Gal4* WPP were measured at 120 hours AED. For each genotype and time point, 9 to 33 wing discs were analyzed. Statistical significance was determined using unpaired *t* tests or one-way ANOVA, as appropriate. n.s., not significant; ****P ≤ 0.0001. The percentage reduction in wing disc volume caused by FBP1 knockdown relative to *UAS-rfp^RNAi^;Lpp-Gal4/rn-Gal4*,*UAS-avl^RNAi^* at 144 and 156 hours AED is indicated above the asterisks. (**D**) Representative confocal images of tumor wing discs from *UAS-rfp^RNAi^;Lpp-Gal4/rn-Gal4*,*UAS-avl^RNAi^* and *UAS-Fbp1^RNAi^;Lpp-Gal4/rn-Gal4*,*UAS-avl^RNAi^* larvae, illustrating OPP incorporation. Scale bar, 100 μm. (**E**) Quantification of OPP levels in tumor wing discs from larvae with or without FBP1. Twelve tumor wing discs per genotype were analyzed, and statistical significance was determined using an unpaired *t* test with Welch’s correction. ***P ≤ 0.001. The percentage reduction in OPP incorporation upon FBP1 knockdown relative to control is indicated above the asterisks. (**F** to **I**) Intratumoral levels of LSP1α (F), LSP2 (G), FBP1 (H), and FBP2 (I) were measured in the following genotypes: *UAS-rfp^RNAi^;Lpp-Gal4/rn-Gal4*,*UAS-avl^RNAi^* and *UAS-Fbp1^RNAi^;Lpp-Gal4/rn-Gal4*,*UAS-avl^RNAi^*. For reference, the 120 hours AED time point, corresponding to pupariation in *UAS-rfp^RNAi^/UAS-gfp^RNAi^;Lpp-Gal4/rn-Gal4* control WPP, was used to determine baseline hexamerin density in wing discs. For each condition and time point, 4 to 19 individual discs or tumors were analyzed.

To determine whether hexamerin uptake is specific to cachectic tumor models or a general feature of tumor development, we examined another type of tumors generated by silencing the *avalanche* gene (*27*). While these tumors originate in the same tissue as the Yki^S168A^ model, their growth kinetics differs: After an initial period of moderate growth, *rn > avl^RNAi^* tumors enter a phase of neoplastic expansion, causing a 2- to 3-day delay at pupariation and culminating in pupal lethality without inducing detectable cachexia (*10*). Immunofluorescence reveals that, similar to Yki^S168A^ tumors, all hexamerins are present at high level in *avl^RNAi^* tumors compared to control wing discs (Fig. 3, A and B). Gene expression confirmed that these proteins were not produced locally by the tumor (fig. S4A). Moreover, hexamerin expression in the fat body was unchanged between control and tumor-bearing larvae (fig. S4B), consistent with their production and secretion by the fat body, followed by their uptake in the tumor. Using quantification of immunofluorescence signals, we observed that hexamerin levels increase steadily during the first 24 to 48 hours and subsequently decline (fig. S4, C to F), a profile similar to the one observed in the Yki^S168A^ model. Silencing *Fbp1* resulted in a complete loss of hexamerins in tumors (fig. S4, C to F), demonstrating that FBP1-dependent import is a general feature of tumor-associated hexamerin uptake, rather than a consequence of fat body wasting caused by cachectic tumors.

Analysis of tumor volume revealed that growth occurred during the period of hexamerin accumulation (Fig. 3C), and the temporal pattern of the growth rate paralleled hexamerin levels (fig. S4G). When FBP1 was knocked down, tumor size was reduced by more than 50% compared to controls (Fig. 3C). Consistently, OPP incorporation assays showed that tumors lacking hexamerins exhibited a 29% decrease in protein synthesis (Fig. 3, D and E), further supporting the notion that storage proteins are critical for sustaining neoplastic growth.

Last, we calculated hexamerin density within *avl^RNAi^* tumors by integrating protein accumulation data with volumetric measurements. This analysis showed that hexamerin density increased during the first 24 hours of the extended larval stage and declined sharply thereafter (Fig. 3, F to I), likely reflecting the progressive depletion of systemic hexamerin stores and the continuous expansion of tumor mass.

Together, these results establish that the uptake of albumin-like proteins both in cachectic (Yki^S168A^) and noncachectic (*avl^RNAi^*) tumors relies on a general FBP1-mediated mechanism that enables tumors to exploit host storage proteins as a source of nutrient.

### Hexamerin uptake by tumors modulates host developmental timing via *dilp8*

Both tumor models used in this study profoundly alter host development. While Yki^S168A^ tumors cause a complete developmental arrest at the end of the larval stage (fig. S1A), *avl^RNAi^* tumor-bearing larvae exhibit a delay of 2 to 3 days before pupariation (*10*).

**Fig. 4.**
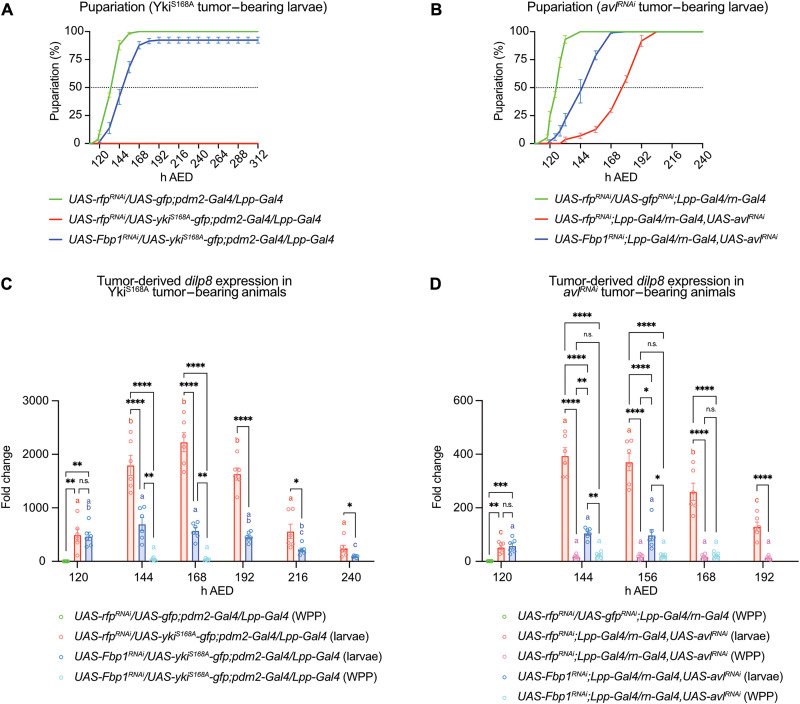
Hexamerins in tumors impair host development through enhanced *dilp8* expression. (**A** and **B**) Pupariation timing curves of larvae bearing Yki^S168A^ (A) or *avl^RNAi^* (B) tumors in the presence (red lines) or absence (blue lines) of FBP1. Additional control genotypes are included (green lines). Time course data represent the mean ± SEM from three vials per genotype (*N* = 3). (**C** and **D**) *dilp8* mRNA levels in dissected wing discs or tumors during the extended larval stage from the following genotypes: *UAS-rfp^RNAi^/UAS-gfp;pdm2-Gal4/Lpp-Gal4*, *UAS-rfp^RNAi^/UAS-yki^S168A^-gfp;pdm2-Gal4/Lpp-Gal4*, and *UAS-Fbp1^RNAi^/UAS-yki^S168A^-gfp;pdm2-Gal4/Lpp-Gal4* (C); and *UAS-rfp^RNAi^/UAS-gfp^RNAi^;Lpp-Gal4/rn-Gal4* (control), *UAS-rfp^RNAi^;Lpp-Gal4/rn-Gal4*,*UAS-avl^RNAi^*, and *UAS-Fbp1^RNAi^;Lpp-Gal4/rn-Gal4*,*UAS-avl^RNAi^* (D). Gene expression was measured by reverse transcription quantitative polymerase chain reaction (RT-qPCR). Bars show the mean ± SEM, with individual replicate values overlaid. Six biological replicates were analyzed per genotype at each time point (*N* = 6). Statistical differences within each genotype across time points were assessed using one-way ANOVA or unpaired *t* tests, as appropriate, and are indicated by color-coded letters above the bars. Comparisons between genotypes at the same time point were also tested using one-way ANOVA or unpaired *t* tests; significance is denoted by asterisks: n.s., not significant; *P ≤ 0.05; **P ≤ 0.01; ***P ≤ 0.001; ****P ≤ 0.0001. Gene expression levels were normalized to *actin*. Developmental timing is reported in hours (h) AED.

Notably, upon *Fbp1* knockdown, the developmental arrest induced by Yki^S168A^ tumors is largely rescued, pupariation occurring with only 11 hours of delay compared to controls (Fig. 4A). This rescue is specific to the loss of FBP1, as silencing other components of the protein storage system did not restore developmental progression (fig. S5A). Similarly, in the *avl^RNAi^* tumor model, blocking hexamerin import into the tumor partially alleviates the developmental delay, allowing earlier pupariation (Fig. 4B and fig. S5B; more controls in fig. S2I).

To investigate whether tumor size influences the ability to pupariate in this context, we measured tumor volumes in *Fbp1*-depleted, Yki^S168A^ tumor-bearing animals. We found that larvae that pupariated [white prepupae (WPP)] showed modestly although significantly smaller tumors than those developmentally arrested (fig. S5C), suggesting that tumor size is not directly correlated with the developmental delay. Previous studies have shown that many types of tumorous imaginal discs secrete Dilp8, an insulin-like peptide that suppresses ecdysone production, thereby delaying metamorphosis (*10*–*14*). However, the dynamics of *dilp8* expression and its regulation by tumor metabolism remain elusive.

We therefore investigated *dilp8* transcription in relation to tumor growth and hexamerin uptake. In Yki^S168A^ tumors, *dilp8* expression increases markedly at 120 hours AED and continues to rise between 144 and 168 hours AED. After 192 hours AED, *dilp8* expression drops despite continued tumor growth (Fig. 4C; see also Fig. 2B). While this pattern does not correlate with the size of the tumor, it is notably superimposable to the tumor growth rate (see fig. S3F), as well as the presence of hexamerins inside the tumor (see fig. S3, B to E). In addition, upon *Fbp1* silencing in the fat body, *dilp8* levels remained at very low levels in animals that pupariated (42% of the population at 144 hours AED and 88% at 168 hours AED; WPP in cyan on Fig. 4C) and reached intermediate levels in larvae that did not (58% of the population at 144 hours AED and 12% at 168 hours AED; larvae in blue on Fig. 4C). These observations indicate that hexamerin uptake is a strong inducer of *dilp8* expression by the tumor and majorly contributes to delaying pupariation. As a control experiment, down-regulation of *Fbp1* specifically in tumors did not affect *dilp8* expression (fig. S5D).

A similar pattern was detected in the *avl^RNAi^* tumor model: *dilp8* expression peaked between 144 and 156 hours AED, followed by a gradual decline despite continued tumor growth (Fig. 4D). As in the Yki^S168A^ model, the rise in *dilp8* expression after 120 hours AED was abolished when hexamerin uptake was blocked by *Fbp1* knockdown in the fat body (Fig. 4D and fig. S5E).

We next asked whether Dilp8 could, in turn, promote hexamerin internalization by the tumor. This possibility was particularly relevant since blocking *dilp8* expression is sufficient to restore pupariation in both Yki^S168A^ (fig. S5F) and *avl^RNAi^* tumor-bearing animals (*10*). For this, we measured *Fbp1* expression in Yki^S168A^ and *avl^RNAi^* tumors upon *dilp8* silencing. In both models, *Fbp1* transcript levels were indistinguishable from control tumors (fig. S5, G and I), indicating that Dilp8 does not control expression of this gene. Consistently, tumor size in *dilp8^RNAi^* tumor-bearing animals was comparable to that of tumors expressing *dilp8* (fig. S5, H and J). Together, our data support the notion that Dilp8 relies downstream of a metabolic switch triggered by hexamerin import and delays developmental progression.

### Hexamerin uptake modulates TORC1 and c-Jun N-terminal kinase signaling in tumor cells

**Fig. 5.**
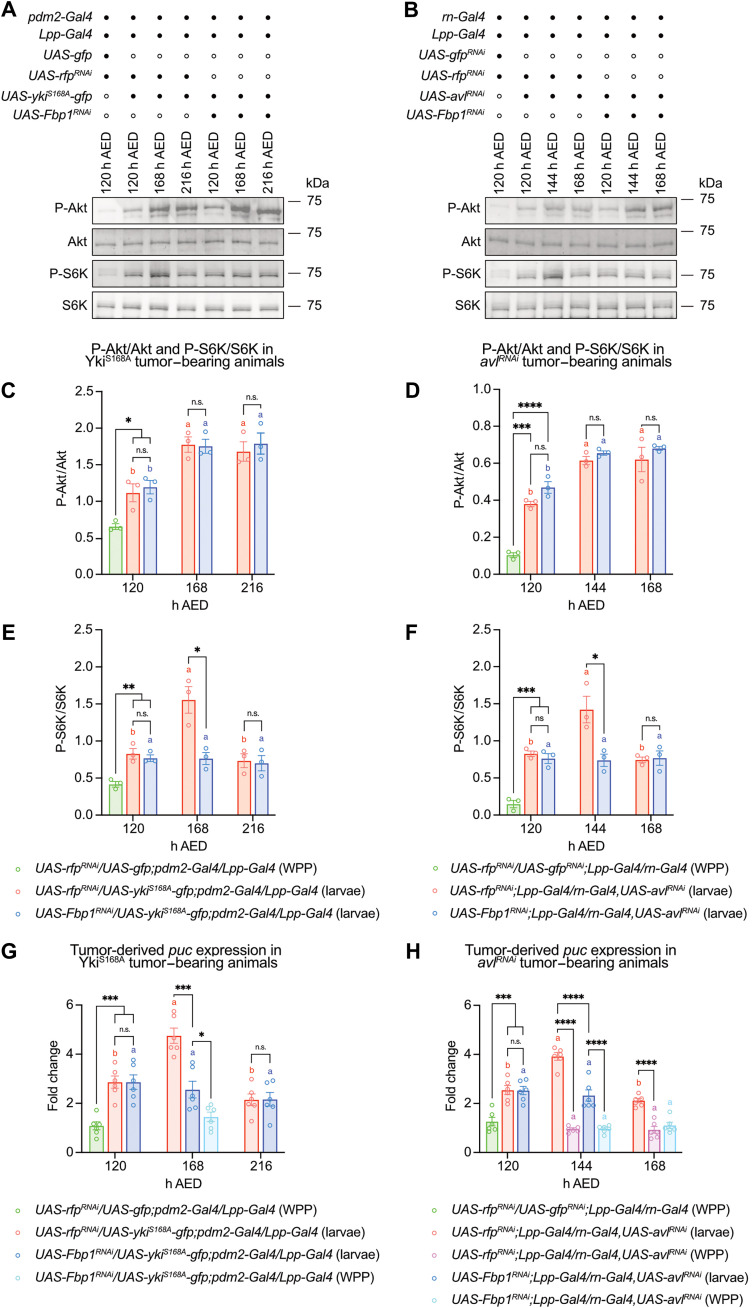
Hexamerin uptake in tumors does not activate Akt but stimulates TORC1 pathway and JNK signaling. (**A** and **B**) Representative Western blots of tumor protein extracts from animals expressing different transgenes at distinct time points (in hours AED). Blots were probed for phospho-Akt (P-Akt), total Akt, phospho-Drosophila p70 S6 kinase (P-S6K), and total S6K, with molecular weight markers indicated on the right. (**C** to **F**) Quantification of the ratios of phosphorylated to total protein: P-Akt/Akt in Yki^S168A^ (C) and *avl^RNAi^* (D) and P-S6K/S6K in Yki^S168A^ (E) and *avl^RNAi^* (F) tumor-bearing animals. Specific genotypes are annotated below the graphs. Band intensities were measured and analyzed using one-way ANOVA or unpaired *t* tests. Statistical significance is indicated as follows: n.s., not significant; *P ≤ 0.05; **P ≤ 0.01; ***P ≤ 0.001; ****P ≤ 0.0001. Each condition includes three biological replicates (*N* = 3). (**G** and **H**) RT-qPCR analysis of *puckered* (*puc*) transcript levels in Yki^S168A^ (G) and *avl^RNAi^* (H) tumor–bearing animals. Specific genotypes and time points are indicated below the graphs. Data are presented as mean ± SEM with individual data points shown. Six biological replicates were included per condition for each time point (*N* = 6). Statistical comparisons across time points within the same genotype were performed using one-way ANOVA or unpaired t tests and are indicated by letters of the same color above the bars. Comparisons between genotypes at matching time points were evaluated similarly and are marked with asterisks: n.s., not significant; *P ≤ 0.05; ***P ≤ 0.001; ****P ≤ 0.0001. Transcript levels were normalized to *actin*, and time points are expressed in hours AED.

To uncover the molecular mechanisms by which hexamerin internalization induces *dilp8* expression, we evaluated the activity of pathways involved in cell growth and stress, namely, insulin, target of rapamycin complex 1 (TORC1), and c-Jun N-terminal kinase (JNK) signaling. Akt phosphorylation, used as a marker for insulin signaling, increased significantly during the initial hours of the extended larval stage in both tumor models and then remained constant (Fig. 5, A to D). The absence of hexamerins did not alter Akt phosphorylation in either Yki^S168A^ or *avl^RNAi^* tumors (Fig. 5, A to D), indicating that insulin signaling is largely insensitive to hexamerin import.

In contrast, TORC1 signaling closely matched both tumor growth rate (see figs. S3F and S4G) and hexamerin dynamics. Phosphorylation of S6 kinase, used as a marker for TORC1 activity, increased in parallel with hexamerin accumulation, reaching maximal levels at 168 hours AED in Yki^S168A^ tumors (Fig. 5, A and E) and at 144 hours AED in *avl^RNAi^* tumors (Fig. 5, B and F). In absence of hexamerins, S6K phosphorylation remained constant throughout the extended larval period (Fig. 5, A, B, E, and F). Therefore, hexamerin uptake in tumor cells supports TORC1-induced tumor growth.

Because JNK was previously shown to strongly induce *dilp8* expression in tumor cells (*10*), we next assessed its levels by measuring expression of *puckered* (*puc*), a target of JNK signaling encoding the JNK phosphatase. In both tumor models, *puc* expression closely followed intratumor hexamerin levels, increasing upon hexamerin uptake but remaining at low levels in absence of hexamerin uptake (Fig. 5, G and H). Together, these results show that tumors exploit host storage proteins in a dual manner: Hexamerins provide amino acids resources leading to the concomitant activation of TORC1 to drive the tumor growth rate and JNK signaling to drive *dilp8* expression.

## DISCUSSION

A central challenge in tumor biology is to understand how proliferating cells meet their nutritional demands while concurrently manipulating systemic signaling for their benefit. In this study, we reveal a mechanism whereby epithelial tumors exploit circulating storage proteins both to fuel their growth and to delay host development.

Taking advantage of their role during *Drosophila* development (*8*), we show that hexamerins produced by the fat body are actively taken up by imaginal disc tumors during the larval stage. Their accumulation in tumors exhibits a biphasic pattern, with levels increasing during the first 24 to 48 hours of tumor growth followed by a decline. This phase of reduced accumulation likely results from both the depletion of circulating stores due to fat body exhaustion and intracellular degradation of internalized hexamerins into amino acids stores. The uptake of these storage proteins in the tumors requires the transporter FBP1, a hexamerin-binding protein previously implicated in regulating hexamerin uptake in fat body cells (*8*). This suggests that the same putative receptor might be used in both tissues for the binding of FBP1 and the subsequent receptor-mediated endocytosis of hexamerin complexes. In both cachectic and noncachectic tumor models, loss of *Fbp1* impairs hexamerin uptake and significantly limits tumor growth. Therefore, hexamerin internalization is not a by-product of systemic wasting but rather appears similar to a general feature promoting tumor growth.

Our findings echo earlier observations that mammalian serum albumins, structurally and functionally analogous to insect hexamerins, are taken up by tumor cells (*2*, *3*, *28*). While the mechanism of albumin uptake in human cancers remains incompletely characterized, it is increasingly clear that, particularly during the early stages of tumorigenesis, albumin does not enter tumor cells solely through passive mechanisms such as macropinocytosis or enhanced permeability and retention. Instead, it requires active import via specific receptors or chaperones, including the secreted protein acidic and rich in cysteine (SPARC) and gp60/albondin in glioblastoma and other cancers (*6*, *29*). This is similar to what we observe in *Drosophila*, where hexamerin uptake by tumors relies on FBP1. The identification of FBP1 as a dedicated hexamerin-associated protein for intracellular uptake in flies provides a genetically tractable platform to investigate how tumors co-opt protein-based nutrient stores and may serve to identify conserved pathways by which tumors exploit host macromolecular resources during early tumor progression.

We also uncover that beyond providing nutritional resources, hexamerin uptake in tumors also promotes systemic effects through the production of Dilp8, a hormone that delays pupariation. This delay provides tumors with additional time to expand before the onset of metamorphosis and the developmental feeding arrest. Blocking hexamerin uptake decreases *dilp8* expression and partially rescues developmental progression. Therefore, hexamerins coordinate internal amino acid stores and endocrine regulations.

**Fig. 6.**
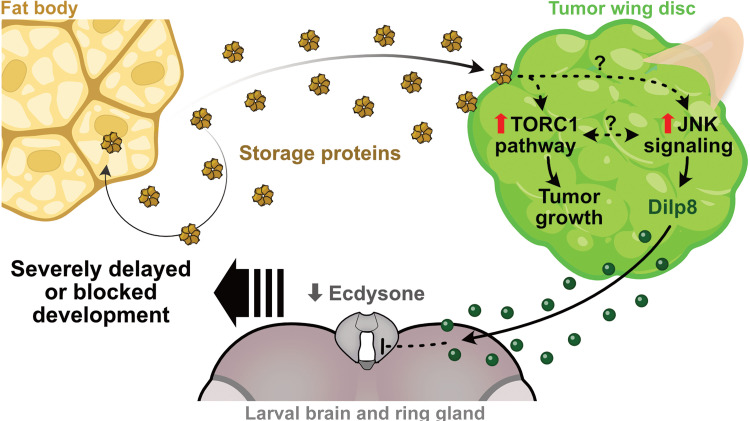
Model summarizing the interplay between tumor hexamerin uptake, *dilp8* expression, and systemic effects on host development. From the pool of storage proteins circulating in the hemolymph, only a small fraction is reabsorbed by the fat body, whereas most is captured by the tumor through FBP1. Hexamerin uptake activates the TORC1 pathway and JNK signaling in tumor cells. JNK, in turn, induces the expression of *dilp8*, which is secreted into the hemolymph and acts on the brain to suppress ecdysone synthesis in the prothoracic gland. Reduced ecdysone levels fail to trigger pupariation, resulting in a strong delay or complete block of host development.

Our findings refine the mechanistic basis of how tumors translate hexamerin uptake into *dilp8* induction. While insulin signaling remains unaffected by suppressing hexamerin import in the tumor, both TORC1 and JNK pathways present a sensible reduction of their activity under these conditions. Although this establishes a functional link between hexamerin uptake and TORC1 and JNK activity levels, the mechanism by which hexamerin import contributes to TORC1 and JNK activation in tumor cells remains elusive and needs to be addressed in further studies. This leads us to propose a model whereby hexamerins in the tumor activate in parallel TORC1 to regulate the growth rate and the stress-response JNK pathway to fine-tune *dilp8* expression and delay development. Alternatively, despite no indication of a direct molecular link between TORC1 activation and JNK signaling in epithelial tumors in *Drosophila*, activation of TORC1 could indirectly induce JNK in response to the metabolic or proliferative stress associated with tumor expansion (see Fig. 6). In contrast, insulin/insulin-like growth factor signaling does not show the dynamics of tumor growth rate or *dilp8* expression and remains largely unaffected by blocking hexamerin uptake, consistent with the possibility that tumors selectively channel hexamerin-derived resources toward TORC1-associated growth and Dilp8-mediated host manipulation.

This endocrine axis parallels the function of relaxins in mammals, a family of hormones related to Dilp8 and known to control tissue remodeling, reproduction, and metabolism (*30*–*32*). Relaxin-like peptides are often up-regulated in human tumors where they contribute to tumor growth, matrix remodeling, and systemic metabolic changes such as cachexia (*33*, *34*). Our findings suggest that the Dilp8/Relaxin pathway may represent a conserved module used by tumors to convert nutrient status into systemic hormonal signals that reprogram host physiology.

These findings open further avenues for exploring how tumors integrate nutrient sensing with hormonal control and raise the possibility that targeting macromolecular nutrient scavenging, such as albumin uptake, may offer therapeutic leverage against tumor progression and associated metabolic syndromes.

In summary, our study shows that tumors in *Drosophila* can act as systemic nutrient sinks, diverting host storage proteins to support their anabolic needs while simultaneously manipulating endocrine circuits to delay development. This dual implication of albumin-like proteins, as both metabolic substrates and regulators of systemic physiology, reveals a conserved evolutionary logic in tumor-host interactions and uncovers fundamental principles of how tumors coordinate metabolic and endocrine manipulation to optimize their progression.

## MATERIALS AND METHODS

### *Drosophila* strains and maintenance

Flies were maintained, and all experiments conducted on a standardized diet consisting of agar (7.5 g/liter), wheat flour (35 g/liter), yeast powder (50 g/liter), sugar (55 g/liter), methyl (25 ml/liter), and propionic acid (4 ml/liter). Experimental conditions were kept constant at 25°C. Both sexes were included in all assays. For precise developmental staging, eggs were collected over a 4-hour window on yeast-supplemented agar plates. L1 larvae were harvested 26 hours after the beginning of egg laying and transferred to vials containing standard food, with 40 larvae per vial. The exact developmental stage or time point used for each analysis is detailed in the relevant sections. The following stocks were obtained from the Bloomington Drosophila Stock Center at Indiana University: *pdm2-Gal4* (#49828), *UAS-gfp* (#39760), *Lpp-Gal4* (#84317), *rn-Gal4* (#7405), *UAS-rfp^RNAi^* (#67852), *UAS-gfp^RNAi^* (#44412), and *UAS-white^RNAi^* (#33623); while the following lines were obtained from the Vienna Drosophila Research Center: *UAS-Lsp1*α*^RNAi^* (#101101), *UAS-Lsp1*γ*^RNAi^* (#38129), *UAS-Lsp2^RNAi^* (#109979), *UAS-Fbp1^RNAi^* (#330200), *UAS-Fbp2^RNAi^* (#33172), *UAS-avl^RNAi^* (#107264), and *UAS-dilp8^RNAi^* (#9420). Other stocks used in this study were *UAS-yki^S168A^-gfp* (*16*) and *rn-Gal4*,*UAS-avl^RNAi^* (*10*)*.*

### Immunostainings, volume reconstruction, and antibody specificity

Wing discs and tumors were dissected from male and female larvae or WPP at the specified developmental stages in phosphate-buffered saline (PBS). Tissues were fixed in 4% formaldehyde (Thermo Fisher Scientific, #28908) for 30 min at room temperature and then washed in PBS with 0.3% Triton X-100 (PBT). After blocking in PBT containing 2% bovine serum albumin (BSA), samples were incubated overnight at 4°C with primary antibodies. The following primary antibodies were used: rat anti-LSP1α (*8*) (1/200), rat anti-LSP2 (1/500) (*8*), guinea pig anti-FBP1 (1/200) (*8*), and guinea pig anti-FBP2 (1/1000) (*8*).

On the following day, tissues were washed, reblocked, and incubated for 2 hours at room temperature with secondary antibodies at 1/200 dilution. These included Goat anti-rat IgG (H + L) Highly Cross-Adsorbed Secondary Antibody, Alexa Fluor 647 (Invitrogen, A21247, Lot 1975526; RRID: AB_141778) and Goat anti-guinea pig IgG (H + L) Highly Cross-Adsorbed Secondary Antibody, Alexa Fluor 568 (Invitrogen, A11075, Lot 1970982; RRID: AB_141954).

Samples were mounted in VECTASHIELD Antifade Medium with 4′,6-diamidino-2-phenylindole (DAPI; Vector Laboratories, #H-1200-10) on glass-bottom CELLview dishes without coverslips to maintain native tissue architecture. Fluorescence imaging was performed on a Zeiss LSM 900 Inverted Laser Scanning Confocal Microscope using the ZEN software (Zeiss). Full tissue volumes were captured by acquiring Z-stacks at 4.27 μm intervals. Image processing and three-dimensional (3D) surface reconstruction were carried out using Fiji and the Imaris software (Oxford Instruments) (*35*), and all conditions, including controls, were processed uniformly.

To assess the specificity of the anti-LSP and anti-FBP antibodies used for immunostaining in tumor tissues, we analyzed the presence of all LSPs and FBPs in tumor cells from animals in which the expression of one gene at a time was silenced (fig. S6). Specifically, *Lsp1*α was silenced in the experiment shown in fig. S6A, *Lsp2* in fig. S6B, *Fbp1* in fig. S6C, and *Fbp2* in fig. S6D.

### Larval tumor growth rate

Instantaneous tumor growth rate (*dV*/*dt*) was estimated at each measured time point by computing the first derivative of a curve fitted to mean tumor volume data. Natural cubic splines were fitted using scipy.interpolate.CubicSpline (bc_type = “natural”; Python 3.12.3, SciPy v1.17.0, NumPy v2.4.2). The first derivative of each spline was evaluated at the original measurement time points to obtain growth rate values aligned with the experimental time axis.

For Yki^S168^ tumors, splines were independently fitted to *UAS-rfp^RNAi^/UAS-yki^S168A^-gfp;pdm2-Gal4/Lpp-Gal4* and *UAS-Fbp1^RNAi^/UAS-yki^S168A^-gfp;pdm2-Gal4/Lpp-*Gal4 data; in both cases, the number of time points was sufficient to reliably constrain the spline and its derivative. For *avl^RNAi^* tumors, the *UAS-rfp^RNAi^;Lpp-Gal4/rn-Gal4*,*UAS-avl^RNAi^* group was analyzed using the same approach. In contrast, only three time points were available for *UAS-Fbp1^RNAi^;Lpp-Gal4/rn-Gal4*,*UAS-avl^RNAi^* tumors, which were insufficient to constrain a cubic spline fit and resulted in unstable derivative estimates. Growth rate was therefore not estimated for these tumors.

Uncertainty in growth rate was estimated by error propagation: Mean volumes were perturbed by ±1 SEM, splines were refit, and derivatives were reevaluated at each time point. The half-range of the resulting values was used as the propagated SEM. Growth rate values and propagated SEMs were plotted as a function of time using GraphPad Prism (version 11; GraphPad Software, San Diego, CA).

### Reverse transcription quantitative polymerase chain reaction

Larvae and WPP were collected at specific times AED. Wing discs or tumors were dissected in cold PBS and immediately flash frozen in liquid nitrogen. Total RNA was isolated using the RNeasy Plus Micro Kit (QIAGEN, #74034) following the manufacturer’s instructions. For each sample, 1 μg of RNA was reverse transcribed using a SuperScript IV VILO Master Mix (Invitrogen, #11756050). The resulting cDNA was used for quantitative polymerase chain reaction (qPCR) on a ViiA 7 system (Applied Biosystems). Each cDNA sample was diluted 1:50 in water and then combined with 10 μM gene-specific primers and Power SYBR Green PCR Master Mix (Applied Biosystems, #4367659). Gene expression was normalized to either *actin* or ribosomal protein *rp49* transcripts. Three independent biological replicates were analyzed, with each sample run in three technical replicates.

Primers used included the following:

actin_For: TCGATCATGAAGTGCGACGT; actin_Rev: ACCGATCCAGACGGAGTACT.

rp49_For: CTTCATCCGCCACCAGTC; rp49_Rev: CGACGCACTCTGTTGTCG.

Lsp1α_For: GAGTACATTGCGATGGGAAAGC; Lsp1α_Rev: CATACGAGCGAAGGCCACAT.

Lsp1β_For: GATCGCCATCGCATTGCTG; Lsp1β_Rev: CCCTGCTTGATGTGGTCCT.

Lsp1γ_For: GCCTGTGTGACTGCCTTTAG; Lsp1γ_Rev: AGAGGCTCATCAATACGGTGA.

Lsp2_For: CTTCCAGCACGTCGTCTACTG; Lsp2_Rev: CCCTGCATATCATCACGGAACA.

Fbp1_For: ATCGTGGCGGCATTGATAAGG; Fbp1_Rev: CGAAGGGTGTCAAAGTCCTG.

Fbp2_For: ATGAATCTGACTGGCATGATCCA; Fbp2_Rev: CCAGGCCATAGACAGAGGACA.

dilp8_For: CGACAGAAGGTCCATCGAGT; dilp8_Rev: GATGCTTGTGCGTTTTG.

puc_For: TCCGGCGGTCTACGATATAGAAA; puc_Rev: AGCAATAGATGCGGGAAAA.

### Western blots

Wing discs, tumors, and fat bodies were collected from animals at defined developmental stages and then homogenized in ice-cold PBS supplemented with Halt Protease & Phosphatase Inhibitor Cocktail (Thermo Fisher Scientific, #78440) using a KIMBLE Dounce tissue grinder (Merck, #D8938). Homogenates were immediately snap frozen in liquid nitrogen. For hemolymph collection, larvae were bled into a drop of ice-cold PBS containing the same inhibitor cocktail. Serum was clarified by repeated centrifugation at 4°C to eliminate cellular contaminants. Protein extracts were prepared from the same number of animals or dissected organs collected at different time points. Samples were mixed with 4× Laemmli Sample Buffer (Bio-Rad, #1610747) containing 10% 2-Mercaptoethanol (Sigma-Aldrich, #M6250) and then boiled at 95°C for 10 min. Proteins were separated by SDS-polyacrylamide gel electrophoresis using Mini-PROTEAN TGX Precast Gels (Bio-Rad, #4569033) in Tris/Glycine/SDS Buffer (Bio-Rad, #1610732) and transferred to 0.2-μm nitrocellulose membranes with the Trans-Blot Turbo system (Bio-Rad, #1704158). Membranes were blocked in tris-buffered saline (Bio-Rad, #1706435) + 0.1% Tween-20 + 5% BSA and then incubated with the following primary antibodies: rat anti-LSP1α (1:1000) (*8*), rat anti-LSP2 (1:5000) (*8*), guinea pig anti-FBP1 (1:1000) (*8*), guinea pig anti-FBP2 (1:5000) (*8*), rabbit anti-phospho-Akt (P-Akt; Cell Signaling Technology, #4060; RRID: AB_2315049; 1:1000), rabbit anti-Akt (Cell Signaling Technology, #9272; RRID: AB_329827; 1:1000), rabbit anti-phospho-Drosophila p70 S6 kinase (P-S6K; Cell Signaling Technology, #9209; RRID: AB_2269804; 1:1000), and guinea pig anti-Drosophila S6K (1:2000) (*36*). Loading controls included rabbit anti-Actin (Sigma-Aldrich, #A2103; RRID: AB_476694; 1:5000) and guinea pig anti-Cv-d (1:1000) (*21*). Horseradish peroxidase-conjugated secondary antibodies (Invitrogen, #31470; RRID: AB_228356; Jackson ImmunoResearch, #111–035-144; RRID: AB_2307391; Invitrogen, #A18769; RRID: AB_2535546. 1:5000) were used with the Clarity Max Western ECL Substrate (Bio-Rad, #1705062) for signal detection. All results are representative of three independent biological replicates. Band intensities were quantified using the Fiji software.

### Hexamerin absolute signal quantification and its density

After staining procedures were completed, tissues were placed in glass-bottom CELLview dishes containing VECTASHIELD Antifade Mounting Medium with DAPI, omitting the use of coverslips. Imaging was carried out using a Zeiss LSM 900 inverted confocal microscope operated via the ZEN software (Zeiss). To reconstruct entire tissue volumes, Z-stack images were collected at 4.27-μm intervals.

To quantify the total hexamerin signal within tumors, we developed a custom Fiji macro [code in the Zenodo repository (https://doi.org/10.5281/zenodo.17661315)]. First, images were converted to TIFF format. Tumor regions were segmented using either green fluorescent protein (GFP) fluorescence or DAPI staining, depending on the genotype. Specifically, for Yki^S168A^ tumors, GFP fluorescence was used as a mask to delineate the tumor area. In contrast, for *avl^RNAi^* tumors, which lacked GFP labeling, DAPI staining was used to define the tumor boundaries. Within each Z-slice, the macro measured (i) the mean gray value of the hexamerin signal within the segmented tumor region and (ii) the area of this region. Because the mean gray value is the sum of the gray values of all the pixels in the selection divided by the number of pixels, multiplying (i) by (ii) yielded the absolute hexamerin signal for that slice. The total signal for the tumor was obtained by summing the absolute values across all slices. This cumulative signal was subsequently normalized to the tissue volume (in cubic micrometers) to compute the hexamerin signal density.

### OPP incorporation assay

Protein synthesis was assessed using the Click-iT Plus OPP Protein Synthesis Assay Kit (Life Technologies, C10457). Tumor discs were dissected in Schneider’s Drosophila medium (Gibco, #21720-024) and incubated with OPP at a final concentration of 20 μM for 30 min. Following incubation, tissues were rinsed in PBS and fixed in 4% formaldehyde for 20 min. Staining was carried out according to the manufacturer’s instructions.

For quantification, a single focal plane image was acquired for each sample. OPP fluorescence intensity was measured using Fiji as the integrated density of the red channel and normalized to the tumor wing disc area, which was defined by the nuclear staining provided in the kit (*37*).

### Food intake

Food intake was measured using a dye-based assay, as previously reported (*38*). Synchronized third-instar larvae were rinsed in PBS, blotted dry with a Kimwipe, and placed on standard fly food containing 1.5% (w/v) blue dye (Erioglaucine Disodium Salt, Merck, #861146) for 1 hour at 25°C. After feeding, larvae were washed in PBS, dried, weighed, flash frozen in liquid nitrogen, and stored at −20°C. For analysis, larvae were homogenized in 20 μl of PBS and spun down at 4°C for 20 min. A 10-μl sample of the supernatant was transferred to a clean tube, and dye levels were quantified by measuring absorbance at 629 nm using a spectrophotometer. Results were then normalized to the corresponding larval mass.

### Wing imaginal disc volume

Wing discs were dissected from larvae at 120 hours AED in ice-cold PBS and then fixed in 4% formaldehyde for 20 min. Tissues were mounted in VECTASHIELD Antifade Medium with DAPI on CELLview glass-bottom culture dishes without coverslips. Imaging was carried out using a Zeiss LSM 900 inverted confocal microscope, and Z-stack images were processed with the Imaris software (*35*).

### Growth curves

Adult females laid eggs for 4 hours on plates containing PBS, 2% agar, and 2% glucose. After 26 hours, 40 first-instar larvae were transferred to tubes with standard fly food and kept at 25°C. To track pupariation timing, larvae were monitored every 2 to 3 hours, and the number entering pupariation was recorded. For eclosion timing, five groups of 40 synchronized WPP per genotype were followed, and newly emerged adults were counted to generate eclosion curves.

### Adult wing area

Adult females of the desired genotype were collected and preserved in ethanol. Wings were dissected and mounted in a 6:5 lactic acid to ethanol solution. Images were captured at 1024 × 768 resolution using a Leica MZ16-FA fluorescence stereomicroscope equipped with a DFC-490 digital camera in bright-field mode (settings: 50% light intensity, 10.5 exposure, 2.3 gain, 152 saturation, 1.2 gamma). Wing area was quantified using a previously established deep learning–based segmentation method (*35*).

## Supplementary Materials

### This PDF file includes:

Figs. S1 to S6 Uncropped Western blot
