# Supplementary material for: Drosophila storage proteins promote both the rate and the duration of tumor growth
Source: Sci Adv. 2026 Jul 3;12(27):eaeb2956. doi: 10.1126/sciadv.aeb2956 (PMC13330821; doi:10.1126/sciadv.aeb2956)
Supplement: Supplementary file 1 — Figs. S1 to S6 Uncropped Western blot [file sciadv.aeb2956_sm.pdf]

Science Advances
AAAS

Supplementary Materials for

Drosophila storage proteins promote both the rate and the duration of tumor growth

Luca Valzania *et al.*

Corresponding author: Luca Valzania, luca.valzania@curie.fr; Pierre Léopold, pierre.leopold@curie.fr

Sci. Adv. **12**, eaeb2956 (2026)
DOI: 10.1126/sciadv.aeb2956

This PDF file includes:

Figs. S1 to S6
Uncropped Western blot

SUPPLEMENTARY MATERIALS
(FIGURES S1-S6 AND CORRESPONDING LEGENDS)

Figure S1

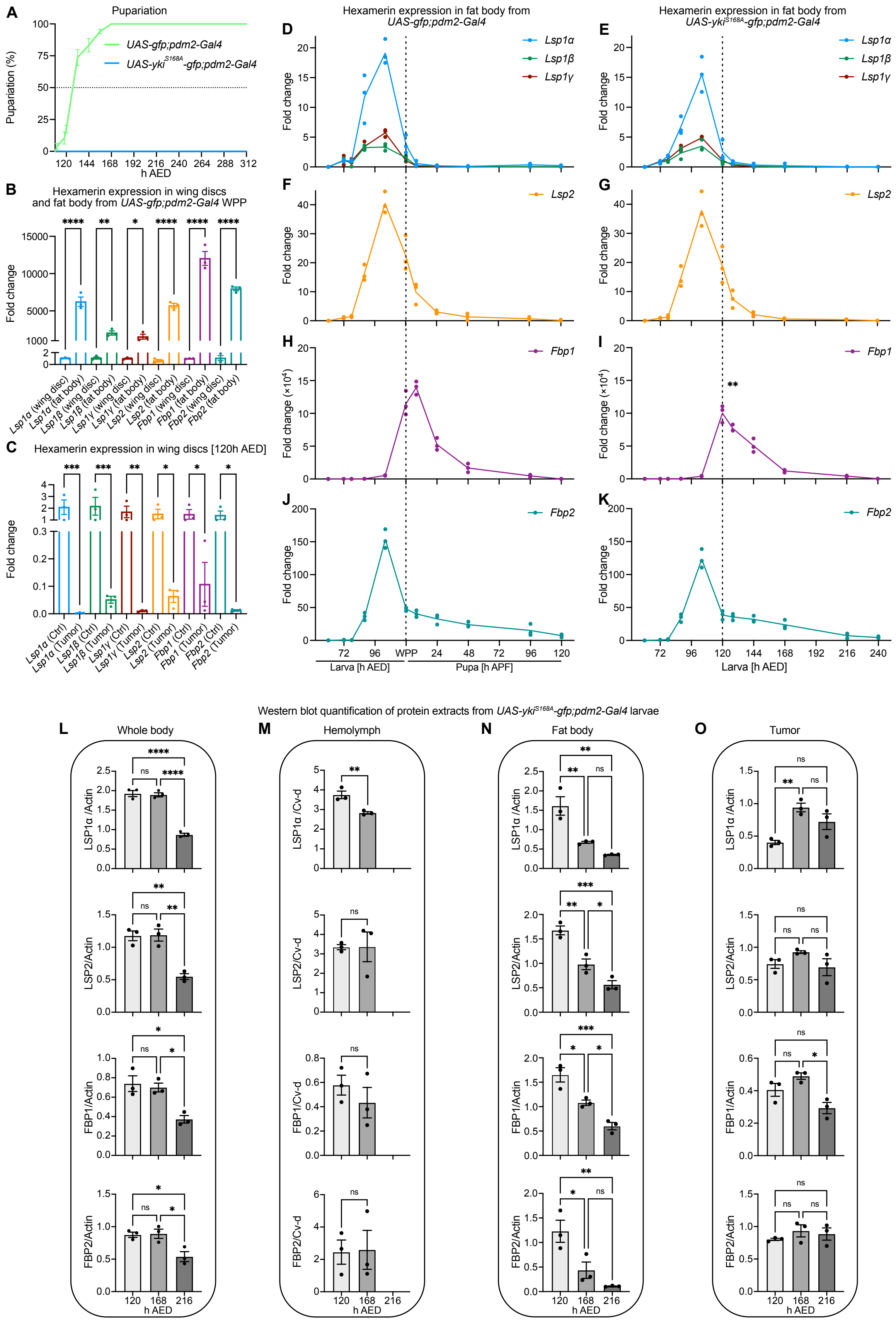

Fig. S1. **Yki^{S168A} tumors scavenge circulating hexamerins without inducing their expression or producing them locally.** (**A**) Timing of pupariation for *UAS-gfp;pdm2-Gal4* and *UAS-yki^{S168A}-gfp;pdm2-Gal4* animals. Mean values ± SEM are plotted over time (x-axis: h AED). For each genotype, N = 3 represents the mean from three separate vials in a single experiment. (**B**) RT-qPCR analysis of *Lsp* and *Fbp* transcripts in wing discs and fat bodies isolated from *UAS-gfp;pdm2-Gal4* white prepupae (WPP). Each data point corresponds to an individual replicate; bars indicate mean ± SEM. Significance was evaluated by unpaired t test. * = $p \leq 0.05$; ** = $p \leq 0.01$; **** = $p \leq 0.0001$. N = 3 replicates per gene. Expression was normalized to *rp49*. (**C**) Transcript levels of *Lsp* and *Fbp* genes in wing discs from *UAS-gfp;pdm2-Gal4* (Ctrl) and *UAS-yki^{S168A}-gfp;pdm2-Gal4* (Tumor) animals at 120h AED, measured by RT-qPCR. Each dot represents an independent replicate; bars show mean ± SEM. Statistical significance was assessed using an unpaired t test. * = $p \leq 0.05$; ** = $p \leq 0.01$; *** = $p \leq 0.001$. N = 3 replicates per gene. Expression levels were normalized to *rp49*. (**D-K**) Developmental expression profiles of *Lsp1α*, *Lsp1β*, *Lsp1γ* (**D**, **E**), *Lsp2* (**F**, **G**), *Fbp1* (**H**, **I**), and *Fbp2* (**J**, **K**) in control (*UAS-gfp;pdm2-Gal4*, left panels) and tumor-bearing (*UAS-yki^{S168A}-gfp;pdm2-Gal4*, right panels) animals, analyzed by RT-qPCR at indicated developmental time points. Dotted lines mark the timing of the larva-to-pupa transition in *UAS-gfp;pdm2-Gal4* animals and the equivalent time point in *UAS-yki^{S168A}-gfp;pdm2-Gal4* animals, which fail to undergo pupariation. Each dot represents a replicate; lines connect mean values across time. Comparisons at each time point between genotypes were performed using Welch's t test. ** = $p \leq 0.01$. N = 3 replicates per genotype for each time point. Time is shown as hours after egg deposition (h AED) and hours after puparium formation (h APF). All expression levels were normalized to *rp49*. (**L-O**) Quantification of LSP1α, LSP2, FBP1, and FBP2 protein levels in whole-body lysates (**L**), hemolymph (**M**), fat body (**N**), and tumor (**O**) tissues of *UAS-yki^{S168A}-gfp;pdm2-Gal4* animals at 120, 168, and 216h AED. Protein levels were normalized to Cv-d in hemolymph samples, and to Actin in whole body, fat body, and tumor samples. Data are shown as individual replicates with mean ± SEM. Statistical significance was determined using Tukey's multiple comparison test. ns = not significant; * = $p \leq 0.05$; ** = $p \leq 0.01$; *** = $p \leq 0.001$; **** = $p \leq 0.0001$. N = 3 replicates per time point. Corresponding western blots are displayed in Fig. 1C-F.

Figure S2

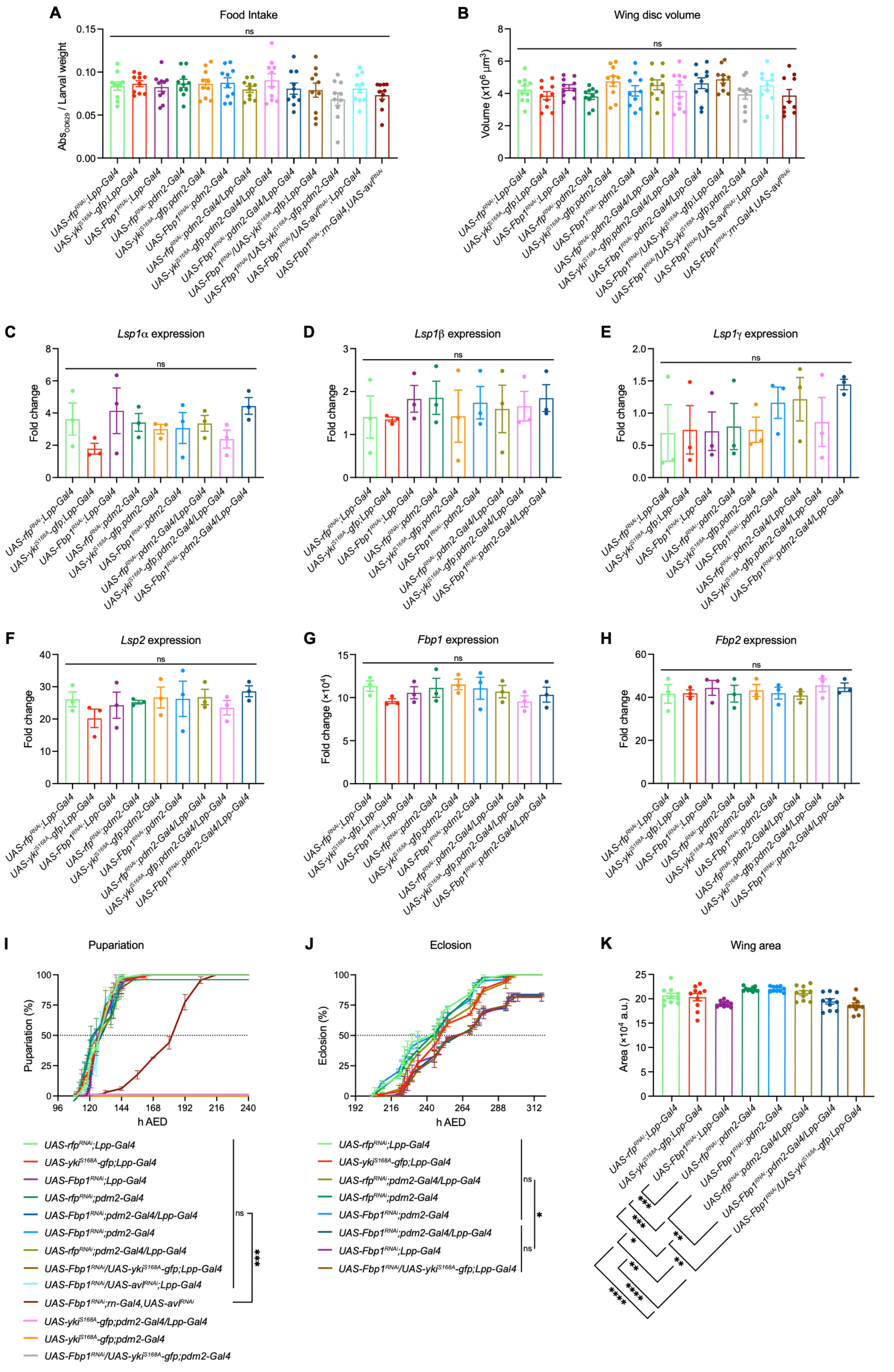
A
Food Intake
Abs$_{OD629}$ / Larval weight
ns
B
Wing disc volume
Volume (x10^6 μm^3)
ns
C
Lsp1α expression
D
Lsp1β expression
E
Lsp1γ expression
F
Lsp2 expression
G
Fbp1 expression
Fold change (×10^4)
H
Fbp2 expression
Fold change
I
Pupariation
Pupariation (%)
h AED
J
Eclosion
Eclosion (%)
K
Wing area
Area (×10^4 a.u.)
UAS-rfp^{RNAi};Lpp-Gal4
UAS-yki^{S168A}-gfp;Lpp-Gal4
UAS-Fbp1^{RNAi};Lpp-Gal4
UAS-rfp^{RNAi};pdm2-Gal4
UAS-Fbp1^{RNAi};pdm2-Gal4/Lpp-Gal4
UAS-Fbp1^{RNAi};pdm2-Gal4
UAS-rfp^{RNAi};pdm2-Gal4/Lpp-Gal4
UAS-Fbp1^{RNAi}/UAS-yki^{S168A}-gfp;Lpp-Gal4
UAS-Fbp1^{RNAi}/UAS-avl^{RNAi};Lpp-Gal4
UAS-Fbp1^{RNAi};rn-Gal4,UAS-avl^{RNAi}
UAS-yki^{S168A}-gfp;pdm2-Gal4/Lpp-Gal4
UAS-yki^{S168A}-gfp;pdm2-Gal4
UAS-Fbp1^{RNAi}/UAS-yki^{S168A}-gfp;pdm2-Gal4

Fig. S2. **Using two Gal4/UAS systems to simultaneously induce wing disc tumors and knock down *Fbp1* do not produce confounding developmental side effects.** (**A**) Larval feeding behavior was assessed by measuring ingestion of Erioglaucine blue dye-supplemented food, normalized to body weight. Data were analyzed using Šidák's multiple comparisons test. No statistically significant differences were observed. N = 10 larvae per genotype. (**B**) Wing disc size was measured in larvae of the indicated genotypes. Individual data points represent disc volumes, with mean ± SEM shown. Statistical comparisons were performed using Šidák's multiple comparisons test. No significant differences were detected. N = 10 wing discs per genotype. (**C-H**) Relative expression levels of hexamerin genes were quantified by RT-qPCR at 120h AED in larvae of the indicated genotypes. All expression levels were normalized to *rp49.* Values represent mean ± SEM. Statistical significance was determined by Tukey's multiple comparisons test. ns = not significant. N = 3 biological replicates per genotype. (**I**) Pupariation timing was tracked in larvae of each genotype, with mean ± SEM across time points plotted as connected lines. Analysis was conducted via two-way ANOVA with multiple comparisons. ns = not significant; *** = $p \leq 0.001$. N = 3, representing the average from three independent vials per genotype. (**J**) Adult eclosion timing was monitored and is presented as mean ± SEM over time, with comparisons made by two-way ANOVA multiple comparisons test. N = 3, each representing the mean from three replicate vials per genotype. ns = not significant; * = $p \leq 0.05$. (**K**) Adult wing area was measured in females of the indicated genotypes. Mean ± SEM is shown, and significant differences were assessed using Šidák's multiple comparisons test. Only significant results are reported: * = $p \leq 0.05$; ** = $p \leq 0.01$; *** = $p \leq 0.001$; **** = $p \leq 0.0001$. N = 10 wings per genotype.

Figure S3

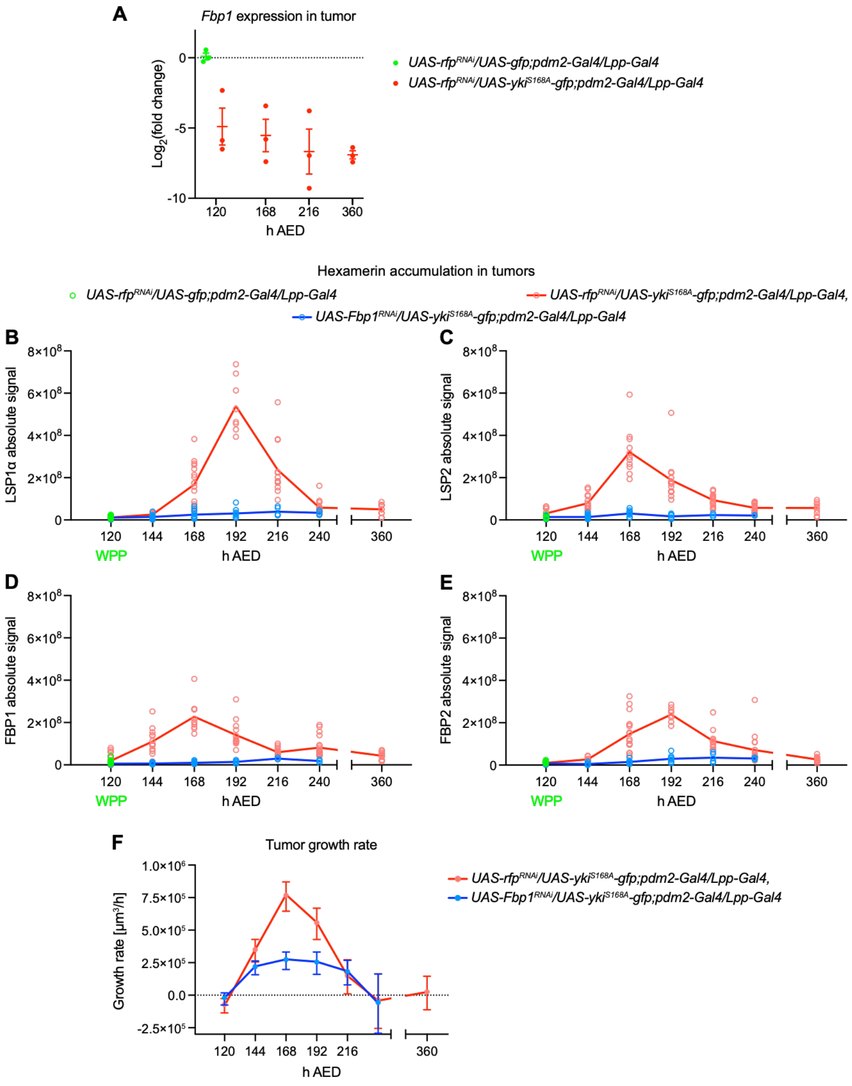

Fig. S3. **Yki^{S168A} tumor progression is marked by dynamic changes in hexamerin levels while *Fbp1* tumor expression remains minimal.** (**A**) Time-course analysis of *Fbp1* expression in wing discs from larvae of the indicated genotypes, measured at multiple developmental stages (x-axis, in hours after egg deposition). Expression values are presented as Log$_2$(fold change), normalized to *rp49*. Each point represents an individual replicate (N = 3 per time point and genotype), with mean ± SEM shown. (**B-E**) Quantification by immunostaining of absolute signal intensity for LSP1α (**B**), LSP2 (**C**), FBP1 (**D**), and FBP2 (**E**) within tumors during the extended last instar larval stage in animals developing tumors either in the presence (*UAS-rfp^{RNAi}/UAS-yki^{S168A}-gfp;pdm2-Gal4/Lpp-Gal4*) or absence (*UAS-Fbp1^{RNAi}/UAS-yki^{S168A}-gfp;pdm2-Gal4/Lpp-Gal4*) of FBP1. Time points (in h AED) are indicated on the x-axis. The 120h AED time point corresponds to pupariation in the control genotype (*UAS-rfp^{RNAi}/UAS-gfp;pdm2-Gal4/Lpp-Gal4*), in which hexamerins were quantified in the wing disc pouch. For each time point, 4 to 18 wing discs/tumors were analyzed per condition. WPP: white prepupa. (**F**) Tumor growth rate was

measured in *UAS-rfp*^{RNAi}*/UAS-yki*^{S168A}*-gfp;pdm2-Gal4/Lpp-Gal4* and *UAS-Fbp1*^{RNAi}*/UAS-yki*^{S168A}*-gfp;pdm2-Gal4/Lpp*-Gal4 larvae at the indicated time points, expressed as h AED. Data are presented as mean ± SEM.

Figure S4

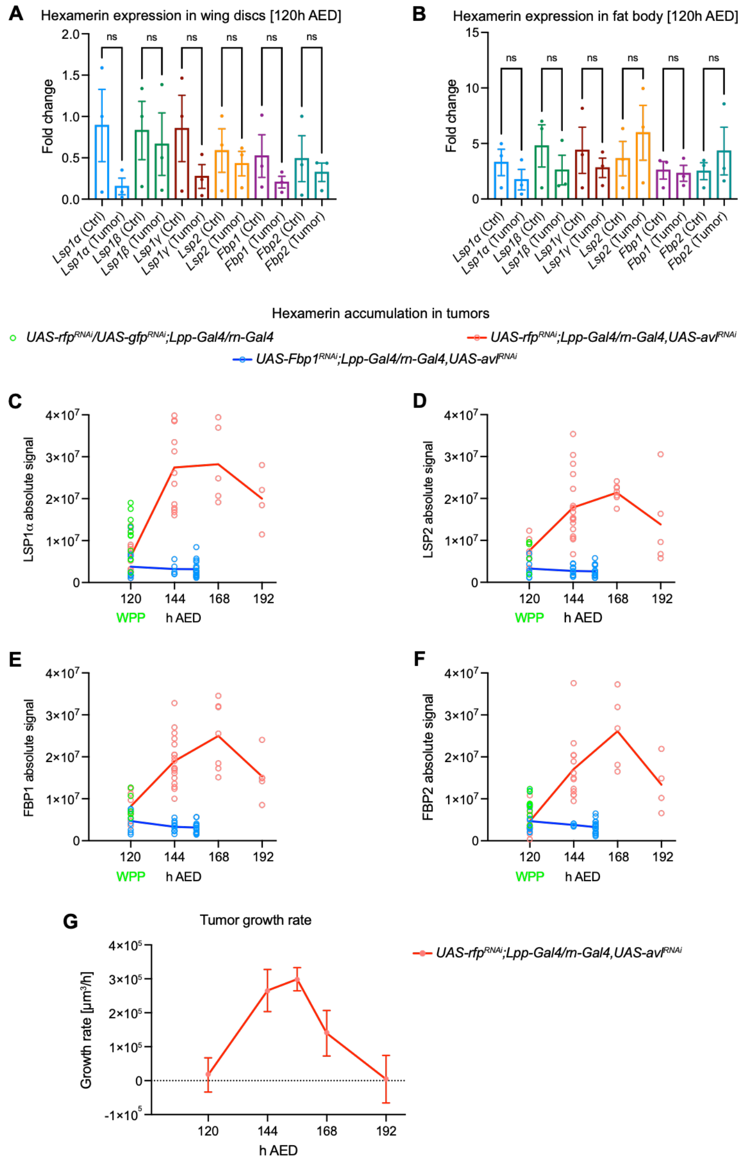

Fig. S4. ***avl^{RNAi}* tumor does not alter hexamerin expression yet promotes their intratumoral accumulation.** (**A**) Relative expression of *Lsp* and *Fbp* genes in wing discs from *UAS-rfp^{RNAi};rn-Gal4* (Ctrl) and *UAS-avl^{RNAi};rn-Gal4* (Tumor) animals at 120h (AED), measured by RT-qPCR. Each dot represents a biological replicate (N = 3 per gene); bars show mean ± SEM. Data were normalized to *rp49*, and statistical comparisons were performed using unpaired t-tests. No significant differences were observed (ns). (**B**) RT-qPCR quantification of *Lsp* and *Fbp* transcripts in fat bodies from *UAS-rfp^{RNAi};rn-Gal4* (Ctrl) and *UAS-avl^{RNAi};rn-Gal4* (Tumor) animals at 120h AED. Individual replicates (N = 3 per gene) are shown, with mean ± SEM. Gene expression was normalized to *rp49*, and unpaired t-tests indicated no significant changes (ns). (**C-F**) Quantification of absolute signal intensity for LSP1α (**C**), LSP2 (**D**), FBP1 (**E**), and FBP2 (**F**) within tumors through immunofluorescence during the extended last instar larval stage. Tumors were analyzed in animals expressing *avl^{RNAi}* either in the presence (*UAS-rfp^{RNAi};Lpp-*

Gal4/rn-Gal4,UAS-avl^{RNAi}) or absence (*UAS-Fbp1*^{RNAi}*;Lpp-Gal4/rn-Gal4,UAS-avl*^{RNAi}) of FBP1. Time points are indicated on the x-axis (in h AED). The 120h AED time point corresponds to pupariation of control animals (*UAS-rfp*^{RNAi}*/UAS-gfp*^{RNAi}*;Lpp-Gal4/rn-Gal4*), in which hexamerin levels were assessed in the wing discs. Between 4 and 19 wing discs or tumors were analyzed per genotype at each time point. WPP: white prepupa. (**G**) Evaluation of tumor growth rate in *UAS-rfp*^{RNAi}*;Lpp-Gal4/rn-Gal4,UAS-avl*^{RNAi} larvae at the indicated time points, expressed as h AED. Data from *UAS-Fbp1*^{RNAi}*;Lpp-Gal4/rn-Gal4,UAS-avl*^{RNAi} animals are not shown in the graph because only three time points were available, which was insufficient to reliably estimate growth dynamics. Additional methodological details are provided in the Materials and Methods section.

Figure S5

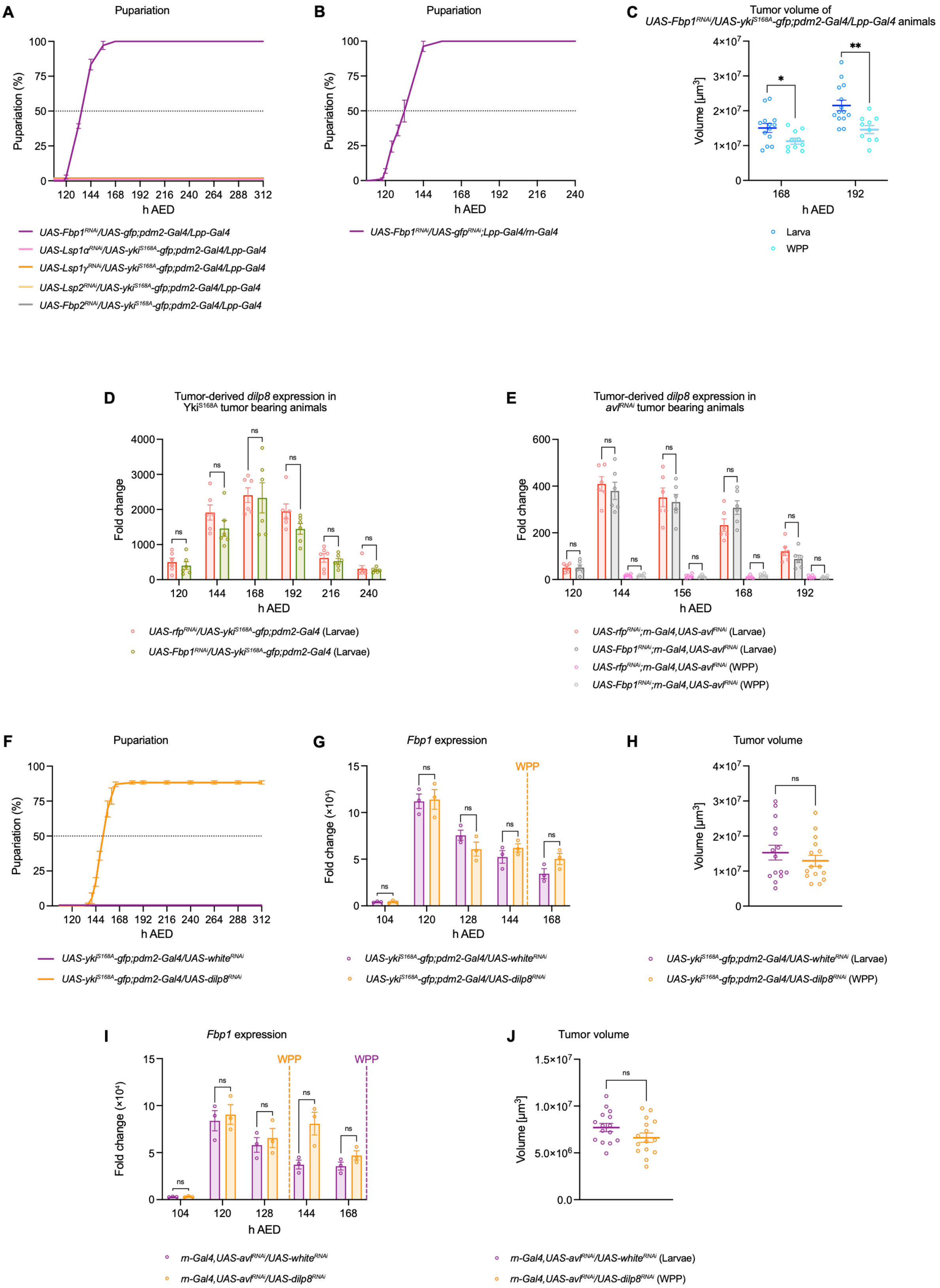

A
Pupariation
Pupariation (%)
h AED
UAS-Fbp1RNAi/UAS-gfp;pdm2-Gal4/Lpp-Gal4
UAS-Lsp1αRNAi/UAS-ykiS168A-gfp;pdm2-Gal4/Lpp-Gal4
UAS-Lsp1γRNAi/UAS-ykiS168A-gfp;pdm2-Gal4/Lpp-Gal4
UAS-Lsp2RNAi/UAS-ykiS168A-gfp;pdm2-Gal4/Lpp-Gal4
UAS-Fbp2RNAi/UAS-ykiS168A-gfp;pdm2-Gal4/Lpp-Gal4
B
Pupariation
Pupariation (%)
h AED
UAS-Fbp1RNAi/UAS-gfpRNAi;Lpp-Gal4/rn-Gal4
C
Tumor volume of
UAS-Fbp1RNAi/UAS-ykiS168A-gfp;pdm2-Gal4/Lpp-Gal4 animals
Volume [µm3]
h AED
Larva
WPP
D
Tumor-derived dilp8 expression in
YkiS168A tumor bearing animals
Fold change
h AED
UAS-rfpRNAi/UAS-ykiS168A-gfp;pdm2-Gal4 (Larvae)
UAS-Fbp1RNAi/UAS-ykiS168A-gfp;pdm2-Gal4 (Larvae)
E
Tumor-derived dilp8 expression in
avlRNAi tumor bearing animals
Fold change
h AED
UAS-rfpRNAi;rn-Gal4,UAS-avlRNAi (Larvae)
UAS-Fbp1RNAi;rn-Gal4,UAS-avlRNAi (Larvae)
UAS-rfpRNAi;rn-Gal4,UAS-avlRNAi (WPP)
UAS-Fbp1RNAi;rn-Gal4,UAS-avlRNAi (WPP)
F
Pupariation
Pupariation (%)
h AED
UAS-ykiS168A-gfp;pdm2-Gal4/UAS-whiteRNAi
UAS-ykiS168A-gfp;pdm2-Gal4/UAS-dilp8RNAi
G
Fbp1 expression
Fold change (×10⁴)
WPP
h AED
UAS-ykiS168A-gfp;pdm2-Gal4/UAS-whiteRNAi
UAS-ykiS168A-gfp;pdm2-Gal4/UAS-dilp8RNAi
H
Tumor volume
Volume [µm3]
UAS-ykiS168A-gfp;pdm2-Gal4/UAS-whiteRNAi (Larvae)
UAS-ykiS168A-gfp;pdm2-Gal4/UAS-dilp8RNAi (WPP)
I
Fbp1 expression
Fold change (×10⁴)
WPP
WPP
h AED
rn-Gal4,UAS-avlRNAi/UAS-whiteRNAi
rn-Gal4,UAS-avlRNAi/UAS-dilp8RNAi
J
Tumor volume
Volume [µm3]
rn-Gal4,UAS-avlRNAi/UAS-whiteRNAi (Larvae)
rn-Gal4,UAS-avlRNAi/UAS-dilp8RNAi (WPP)

Fig. S5. **Dilp8 delays host development in response to hexamerin-driven tumor growth but does not induce *Fbp1* expression or increase tumor size.** (**A**) Timing of pupariation in larvae of the indicated genotypes. These data are derived from the same experimental cohort shown in Fig. 4A. Line graphs represent the mean pupariation percentages ± SEM at various time points (x-axis: h AED). For each genotype, N = 3 corresponds to the average of three independent vials from a single experiment. (**B**) Pupariation curve of *UAS-Fbp1^{RNAi}/UAS-gfp^{RNAi};Lpp-Gal4/rn-Gal4* animals, based on the same dataset shown in Fig. 4B. Mean values ± SEM are plotted over time (x-axis: AED, in hours). N = 3 represents the mean from three separate vials in a single experiment. (**C**) Quantification of tumor volume in *UAS-Fbp1^{RNAi}/UAS-yki^{S168A}-gfp;pdm2-Gal4/Lpp-Gal4* larvae (blue) and white prepupae (WPP, cyan) at 168h and 192h AED. Tumor sizes were compared between larval and white prepupal stages using unpaired t-tests at each time point. * = $p \leq 0.05$; ** = $p \leq 0.01$. For each time point, a total of 10 to 14 tumors were measured per condition. (**D**) Measurement of *dilp8* transcript abundance in tumor discs isolated during the extended larval stage from the following genotypes: *UAS-rfp^{RNAi}/UAS-yki^{S168A}-gfp;pdm2-Gal4* and *UAS-Fbp1^{RNAi}/UAS-yki^{S168A}-gfp;pdm2-Gal4*. Transcript levels were determined by RT-qPCR. Bars represent the mean ± SEM, and individual replicate values are overlaid. Each genotype was assessed in six independent biological replicates per time point (N = 6). Statistical comparisons at identical time points were performed using unpaired t-tests; ns indicates no significant difference. Data were normalized to *actin* transcript levels. Developmental time is indicated in hours after egg deposition (h AED). (**E**) RT-qPCR quantification of *dilp8* mRNA in the following tumor genotypes: *UAS-rfp^{RNAi};rn-Gal4,UAS-avl^{RNAi}* and *UAS-Fbp1^{RNAi};rn-Gal4,UAS-avl^{RNAi}*. Data are presented as mean ± SEM with individual replicate points shown. Six independent samples were analyzed per genotype and time point (N = 6). Comparisons between genotypes at the same developmental stage were made using unpaired t-tests; ns denotes not significant. Transcript levels were normalized to *actin*. Time points are indicated in h AED, with WPP referring to the white prepupa stage. (**F**) Onset of puparium formation in larvae carrying the specified genetic combinations. Curves display the proportion of individuals that had pupariated at each sampled developmental time (x-axis, h AED). Values represent means ± SEM. For every genotype, N = 3 indicates that data derive from three separate vials analyzed within one experimental run. (**G**) Relative abundance of *Fbp1* transcripts in animals with either the *UAS-yki^{S168A}-gfp;pdm2-Gal4/UAS-white^{RNAi}* or the *UAS-yki^{S168A}-gfp;pdm2-Gal4/UAS-dilp8^{RNAi}* genotype. mRNA levels were quantified by RT-qPCR, normalized to *rp49*, and are presented as mean ± SEM with individual biological replicates plotted. Three independent samples were examined for each genotype at each time point (N = 3). Unpaired t-tests were applied for pairwise comparisons at equivalent developmental stages; ns denotes the absence of a significant difference. Developmental time (h AED) is indicated along the x-axis. The dashed vertical marker corresponds to the developmental point at

which 50% of *UAS-yki*^{S168A}*-gfp;pdm2-Gal4/UAS-dilp8*^{RNAi} animals reached the white prepupal (WPP) stage. (**H**) Tumor burden assessed in *UAS-yki*^{S168A}*-gfp;pdm2-Gal4/UAS-white*^{RNAi} larvae and in *UAS-yki*^{S168A}*-gfp;pdm2-Gal4/UAS-dilp8*^{RNAi} animals at the WPP stage, quantified at the moment when WPP individuals of the latter genotype first appear. Tumor dimensions were compared between larval and WPP stages using unpaired t-test; ns indicates no statistically significant change. 15 tumors were evaluated per genotype. (**I**) *Fbp1* transcript quantification in *rn-Gal4,UAS-avl*^{RNAi}*/UAS-white*^{RNAi} and *rn-Gal4,UAS-avl*^{RNAi}*/UAS-dilp8*^{RNAi} animals. Expression levels were measured via RT-qPCR, normalized against *rp49*, and summarized as mean ± SEM with individual replicate points displayed. Each genotype was analyzed in three independent biological replicates at each developmental time (N = 3). Pairwise comparisons at identical time points used unpaired t-tests; ns indicates non-significance. Time after egg deposition (h AED) is indicated. Dashed reference lines mark the timing at which half of the animals of each genotype entered the WPP stage. (**J**) Tumor size measurements in *rn-Gal4,UAS-avl*^{RNAi}*/UAS-white*^{RNAi} larvae and *rn-Gal4,UAS-avl*^{RNAi}*/UAS-dilp8*^{RNAi} individuals at the WPP stage, quantified at the developmental time when WPP individuals of the latter genotype emerge. Comparisons between larval and WPP tumor volumes were performed using unpaired t-test; ns denotes a lack of significant difference. 15 tumors were analyzed for each genotype.

Figure S6

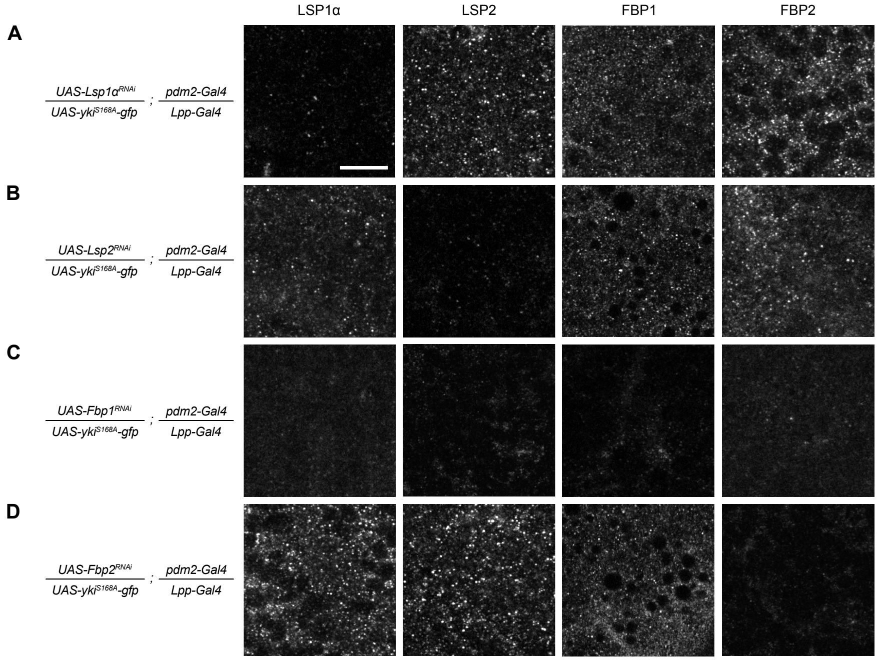

Fig. S6. Validation of antibody specificity for LSPs and FBPs immunostaining in tumor tissues. Confocal images of tumors from animals in which the expression of a single hexamerin gene was silenced are shown. The genotype of each sample is indicated on the left, and the antibody used for immunostaining is shown on the top. Tumors from *Lsp1α*^{RNAi} (**A**), *Lsp2*^{RNAi} (**B**), *Fbp1*^{RNAi} (**C**), and *Fbp2*^{RNAi} (**D**) animals stained with anti-LSP1α, anti-LSP2, anti-FBP1, and anti-FBP2 antibodies. Scale bar = 10µm.

Unedited and uncropped anti-LSP1α western blot corresponding to Fig. 1C, E, F.

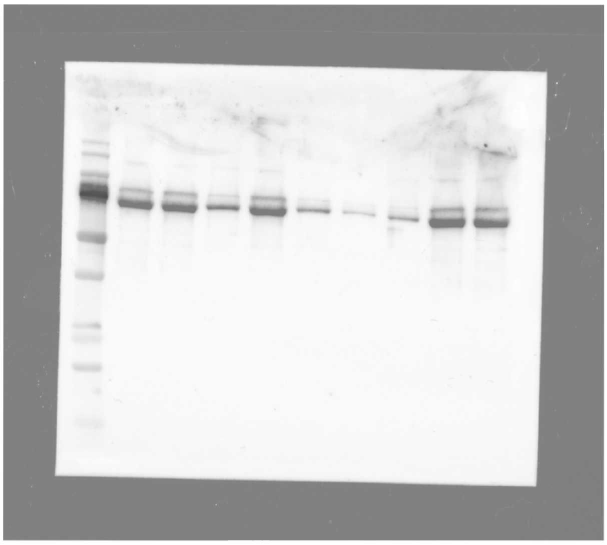

From left to right, lanes contain the molecular weight marker, whole-body protein extracts at three time points (Fig. 1C), fat body protein extracts at three time points (Fig. 1E), and tumor protein extracts at the three analyzed time points (Fig. 1F).
